# ATM-Mediated Transcriptional and Developmental Responses to γ-rays in *Arabidopsis*

**DOI:** 10.1371/journal.pone.0000430

**Published:** 2007-05-09

**Authors:** Lilian Ricaud, Caroline Proux, Jean-Pierre Renou, Olivier Pichon, Sylvain Fochesato, Philippe Ortet, Marie-Hélène Montané

**Affiliations:** 1 CEA, DSV, Institut de Biologie Environnementale et de Biotechnologie (iBEB), Service de biologie végétale et de microbiologie environnementales (SBVME), Cadarache, Saint Paul-lez-Durance, France; 2 Unité de Recherche en Génomique Végétale, UMR INRA 1165 - CNRS 8114 - UEVE, Evry, France; University of Leeds, United Kingdom

## Abstract

ATM (*Ataxia Telangiectasia Mutated*) is an essential checkpoint kinase that signals DNA double-strand breaks in eukaryotes. Its depletion causes meiotic and somatic defects in *Arabidopsis* and progressive motor impairment accompanied by several cell deficiencies in patients with ataxia telangiectasia (AT). To obtain a comprehensive view of the ATM pathway in plants, we performed a time-course analysis of seedling responses by combining confocal laser scanning microscopy studies of root development and genome-wide expression profiling of wild-type (WT) and homozygous ATM-deficient mutants challenged with a dose of γ-rays (IR) that is sublethal for WT plants. Early morphologic defects in meristematic stem cells indicated that AtATM, an Arabidopsis homolog of the human ATM gene, is essential for maintaining the quiescent center and controlling the differentiation of initial cells after exposure to IR. Results of several microarray experiments performed with whole seedlings and roots up to 5 h post-IR were compiled in a single table, which was used to import gene information and extract gene sets. Sequence and function homology searches; import of spatio-temporal, cell cycling, and mutant-constitutive expression characteristics; and a simplified functional classification system were used to identify novel genes in all functional classes. The hundreds of radiomodulated genes identified were not a random collection, but belonged to functional pathways such as those of the cell cycle; cell death and repair; DNA replication, repair, and recombination; and transcription; translation; and signaling, indicating the strong cell reprogramming and double-strand break abrogation functions of ATM checkpoints. Accordingly, genes in all functional classes were either down or up-regulated concomitantly with downregulation of chromatin deacetylases or upregulation of acetylases and methylases, respectively. Determining the early transcriptional indicators of prolonged S-G2 phases that coincided with cell proliferation delay, or an anticipated subsequent auxin increase, accelerated cell differentiation or death, was used to link IR-regulated hallmark functions and tissue phenotypes after IR. The transcription burst was almost exclusively AtATM-dependent or weakly AtATR-dependent, and followed two major trends of expression in *atm*: (i)-loss or severe attenuation and delay, and (ii)-inverse and/or stochastic, as well as specific, enabling one to distinguish IR/ATM pathway constituents. Our data provide a large resource for studies on the interaction between plant checkpoints of the cell cycle, development, hormone response, and DNA repair functions, because IR-induced transcriptional changes partially overlap with the response to environmental stress. Putative connections of ATM to stem cell maintenance pathways after IR are also discussed.

## Introduction

DNA damage recovery is key to cell life because maintaining genome integrity is critical for cell and organism health and reproduction. Therefore, identifying the genetic and biochemical processes leading to tuned DNA repair and cell recovery after damage is for understanding cell function and survival [1]. A large spectrum of DNA lesions occur during physiologic processes or in the presence of toxic external agents, such as IR, which induces clusters of damage including oxidized bases, abasic sites, interstrand crosslinks, single-strand breaks (SSBs), and double-strand breaks (DSBs). When left unrepaired or misrepaired, such lesions usually cause cell death, cancer, or genetic diseases [2]. DNA lesions are repaired by several different mechanisms involving numerous mechanistically and functionally diverse DNA repair protein superfamilies [3]. Ionizing radiation-induced DNA lesions [4], such as oxidized bases and SSBs, are repaired by base and nucleotide excision or encompassed by translesion synthesis. Double-strand breaks activate either the predominant non-homologous-end-joining (NHEJ) repair, which joins free DNA ends and is DNA-protein kinase-dependent in mammals, or homologous recombination repair (HR), which utilizes sister chromatids as a source of undamaged DNA templates for homologous pairing of DNA sequences [5], [6]. Joined repair mechanisms are not mutually exclusive due to the complex processing of lesions such as interstrand crosslink [7] or multiple base lesions that can be processed through subpathways for NHEJ [8], and can be species-specific. DNA lesions trigger signalling cascades through activating checkpoint proteins that stop or delay cell cycle progression, thus allowing DNA repair to take place through various pathways (i.e., HR mainly in the S-G2 phase through inhibition of replication, and NHEJ mainly in the G0-G1 phase) [9]–[11]. The Ataxia Telangiectasia Mutated (ATM) kinase is an essential checkpoint protein that is specifically activated by DNA DSBs and not by SSBs, even at numbers that relax chromatin supercoiling [12]. ATM kinase also mediates cell cycle checkpoints and DSB repair by HR or NHEJ, depending on the DSB end structure and on the cell cycle at the time of damage [13], [14]. Accumulating evidence in yeast and human cells indicates that DSB ends are sensed directly by the MRN protein complex (MRE11, RAD50, NBS1), which binds DNA, unwinds the ends, and recruits ATM *via* NBS1, a process that correlates with ATM activation. The respective role of MRN proteins and other factors depending on the chromatin alteration in the two-step mechanism of ATM stimulation, however, is not yet fully understood [15]–[18]. Activated ATM kinase activates a checkpoint response, including subsequent aggregation of DNA repair proteins, and phosphorylates a large number of substrates depending on the number of DSBs [17], [19], [20]. The 30 to 50 ATM phosphorylation targets so far reported include proteins involved in DNA repair (BRCA1; the DNA-metabolizing proteins Mre11 and NBS1) and in checkpoint control and apoptosis (CHK1 and CHK2, p53). These phosphorylation targets activate cyclin-dependent kinase (CDK) inhibitor p21, and inhibit cyclinA/cyclinE/CDK2 complexes and Polo-kinase, making ATM the master coordinator of G1/S, intra-S, and G2-M cell cycle transitions. Activated CHK2 and facilitators such as BRCA1 drive the phosphorylation of downstream substrates as well as upstream ATM substrates in a highly ordered network [20], [21]. Other ATM targets, such as the telomere factors TRF1 and TRF2, regulators of translation initiation, and DNA replication initiation proteins illustrate the wide variety of cellular functions that are served by ATM to maintain genome integrity in tissues. The role of ATM in the self-renewal capacity of hematopoietic stem cells has led to further exploration of other potential ATM-dependent cellular processes such as cell growth, survival, and anti-tumor immune surveillance [22]–[25]. Indeed, impairment of the human ATM kinase leads to an early onset, progressive, neurodegenerative disorder that is transmitted as an autosomal recessive disorder. AT-patients are hypersensitive to DNA damage and are susceptible to cancer, immunodepression, premature aging, progressive cerebellar ataxia, and oculocutaneous telangiectasia [26]. AT-cells fail to survive DNA damage because of impaired signalling to DNA damage checkpoints and a characteristic inability to arrest DNA synthesis after irradiation, as well as from the inability to repair a small proportion of DSBs [27]. Approximately 90% of the DSBs are repaired faster in AT-cells than in wild-type (WT), except for the so called “slow repair kinetics” DSBs [28]. Like AT-cells, cells deficient in the ARTEMIS nuclease [29], a conserved component of NHEJ phosphorylated by ATM after irradiation, fail to repair the same fraction of DSBs. It has been suggested that ATM directs the processing of the ARTEMIS-dependent hairpin-capped ends towards NHEJ and possibly HR, depending on the presence of sister chromatids. Together these findings illustrate the crucial role of the nuclear serine-threonine kinase ATM in signaling DSBs and in coordinating the complex network of broad cellular functions required to recover from radiation insult.

Another phosphatidyl inositol 3-kinase-like kinase family member, the ATR kinase (*ATM and Rad3-related*), which has an essential function in early mammalian development, has a key role in the checkpoint response to replicative stress and DNA damage caused by alkylating agents or UV-induced DNA lesions [30]–[32]. ATR kinase inhibits cell entry into mitosis and controls premature chromatin condensation, a hallmark of mammalian cells, which begin mitosis before completing DNA replication [33]. ATR is recruited to replication protein A (RPA)-coated single-stranded DNA (ssDNA) by ATR-interacting protein, which is present either during replication as the helicase melts the DNA template and Okazaki fragments are synthesized and joined, or when bulky DNA lesions such as pyrimidine dimers occur. When loaded close to DNA lesions by RAD17 onto the 9-1-1 complex (RAD9-RAD1-HUS1), activated ATR blocks replication and phosphorylates downstream substrates, leading to cell cycle arrest. At DSB sites, the MRN complex proteins activate ATM-damaged DNA and the ssDNA overhangs that are generated are then coated by an RPA forming a nucleofilament on which ATR and the 9-1-1 complex are subsequently loaded, resulting in replication fork arrest. In this case, the checkpoint kinase CHK1, which is usually considered to be an exclusive phosphorylation substrate of ATR in the absence of DSBs, requires the combined action of both ATM and ATR [34]–[37]. ATR also acts to repair by, for example, controlling the ubiquitination of DNA repair-associated proteins such as FANCD2, a crucial modification required for FANCD2 localization to the DNA damage foci close to the recombinational repair protein BRCA2 [38]. This explains why ATR colocalizes in irradiated cells arrested at the S/G2 phases with ATM and the recombinosome proteins that include proteins involved in HR and replication, into subcompartmentalized complexes at DSB sites surrounded by phosphorylated γ-H2AX chromatin zones, a DSB marker [39]. Even in the absence of external DNA damage, the combined action of both kinases is hypothesized to mediate replication regulation, sensing ongoing replication, and in turn downregulating close and distal origins, and replicons through inhibiting the S-phase kinases and the replicative MCM helicase complex [40]. Indeed, ATR and ATM kinases prevent the accumulation of DSBs and promote the restart of collapsed replication forks [41]. The spatiotemporal dynamics of DNA repair and checkpoint proteins that cooperate in large complexes to survey genome integrity in eukaryotic cells suggests that one of the roles of the checkpoint response is to reorganize the protein composition of such complexes through the posttranslational modifications of key components with the aim of rapidly reacting to DNA damage [42]. Many proteins of the DNA damage response are involved in both checkpoint and repair [43] and their access to DNA damage sites largely depends on the dynamics of the DNA and chromatin compaction/relaxation states [44], [45].

ATM-deficient plants show no defects at the vegetative level, but have fertility defects, are hypersensitive to IR and methyl methane sulfonate, fail to early-upregulate DNA repair genes RAD51 and Poly (ADP-ribose)-polymerase1 (PARP1), and delay the upregulation of the NHEJ component ligase IV [46]. ATR-deficient plants do not show somatic or meiotic defects, a divergent characteristic compared to humans. ATR-deficient plants are moderately sensitive to IR and the number of G2-arrested cells is partially influenced as observed 8 hours after IR [47]. Among the earliest cytologic events triggered by IR in plants, γ-H2AX foci have only been studied in M-phase nuclei, and occur in a dose-and time-dependent manner [48]. At the molecular level, DNA DSBs are visualized in WT plantlets [49]) treated with bleomycin, a genotoxin that partially mimics IR. Genome sequencing and systematic insertional mutagenesis have increased the number of plant DNA repair functions identified by reverse genetics, or by means of sterile mutants or altered somatic recombination rates [50], [51] but does not yet compare with yeast and mammalian data [52], and therefore plant DNA metabolism and DNA damage- signaling pathways are still poorly characterized in plants at the biochemical level. Complementary approaches such as proteome analysis of meiotic cells and genome wide-transcript profiling analysis will help to characterize those functions in plants [53]. In response to genotoxins, there is strong expression of the G2/mitotic cyclin B1;1 and conserved DNA repair genes such as AtRAD51, AtBRCA1, or AtPARP1 (sometimes up to more than 100 fold [46], [49], [54]–[56]), making plant transcript profiling very informative for identifying DNA damage responses. Similarly, the constitutive expression of DNA metabolism genes in mutants defective in chromatin metabolism [57], [58] are indicators of DNA repair pathways. This is in clear contrast to studies in mammals, in which transcriptional induction of these genes is usually either weakly documented or far much less active than is the posttranslational modification of the encoded protein [59], [60]. In addition, the presence of checkpoints at meiosis and/or after DNA damage is still under debate in plants, although the cell cycle is relatively well characterized [51]. Here, we analysed transcriptional and developmental changes occurring after IR in WT and *atm* to characterize the extent of the role of ATM in the DNA damage response pathway in plants, and the link between molecular and tissue phenotypes.

## Results

### Sublethal IR promotes an early and transient arrest of cell division that is differentially relaxed, auxin increase, and vascular cell death

For several hours or days after heavy IR of seeds and seedlings, developmentally arrested seedlings called “gamma plantlets” are blocked outside of M phase at G2 and/or at G1/S as measured by flow cytometry and cyclin B1;1-GUS activity [47], [61]–[63]. Our aim was to describe the sequence of events occurring in roots from the time of IR to the time of growth restart in WT seedlings irradiated with a sublethal dose of 100Gy γ-rays. Those conditions trigger early maximal upregulation of transcripts [54] and transiently delay seedling growth [46], enabling us to study the link between transcriptional change and subsequent development. Therefore, the effect of IR on development was analyzed in live seedlings carrying growth-associated markers. The lines included (i) cyclin *AtCYCB1;1-*green fluorescent protein (GFP), which marks cells arrested in late S through early M phases [64], [65], and therefore activation, persistence, and relaxation of IR-induced cell division arrest; (ii) histone *AtH2B-*yellow fluorescent protein (*YFP*), a marker of chromatin organization, DNA content, and nuclear morphology, allowing us to visualize the relative evolution of cell DNA content in the organ [66], [67]; and (iii) DR5-GFP, a marker of auxin response which typically can be used to reflect changes in auxin content and distribution which are key regulators of organ growth [68], [69].

**Figure 1:**
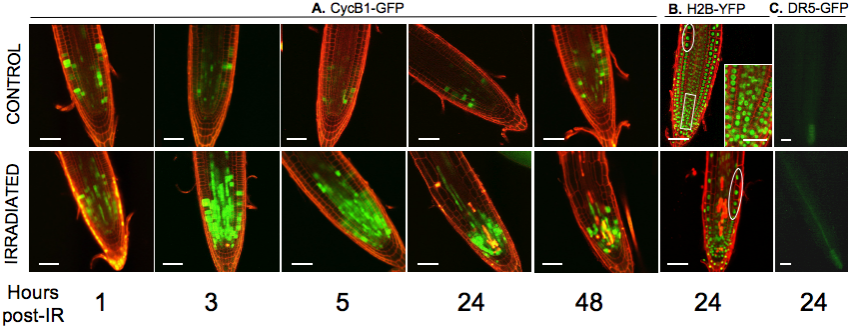
Root tip morphology and expression of fluorescent markers of WT seedlings after IR. CLSM optical sections showing root tip morphology and expression of cell cycle, nucleus size, and auxin markers. Superimposition of images showing GFP and YFP distribution (green) and cell outlines stained with PI (red). Dying cells are bright red due to PI uptake. A.: Cyclin B1;1 time-course after IR. After 24 h, loss of the transition zone results in a reduced meristematic zone still containing division-inactive cells next to a large zone of differentiated cells without CYCB1;1 expression. B.: Distribution of Histone H2B-YFP-stained nuclei. In controls, the rectangle (enlarged in insert) shows (from bottom to top) typical examples of condensed chromatin of G2-like nuclei, early prophase (loss of spherical shape) and metaphase plates. Circled areas show large differentiating cells with high DNA content far from root tip in controls and close to root tip after IR. C.: Epifluorescence microscope image showing expression of DR5-GFP in living root tips 24 h after IR. (Bars = 50 µm).

Stereomicroscopic observation and optical sectioning of living seedling roots using confocal laser scanning microscopy indicated that the number of cells accumulating cyclin B1-GFP in the whole meristematic zone strongly increased during the first hour post-IR with a peak at 3 to 5 h (Fig. 1-A), remained constant for approximately 24 to 52 h, and then decreased towards the non-irradiated root levels (Fig. S1). This finding indicated that cell division was delayed from late S or late G2, i.e., the G2/M transition for most meristematic cells, as previously reported in gamma plantlets [47], [62]. The arrest was earlier and transient, however, consistent with a sublethal IR dose. Not all cells accumulated CYCB1;1, suggesting that a subpopulation of cells arrested at another cell cycle phase, i.e., at G1 and early S*.* One day after IR, the meristematic zone marked by CYCB1;1-GFP was nearly half that observed a couple of hours after IR and was restricted to the region close to the quiescent center (QC) (Fig. 1-A). The cells of the meristematic zone that have lost CYCB1;1 fluorescence, were abnormally elongated and enlarged in every tissue, and were immediately adjacent to a set of cells including stem cells that stayed arrested longer. This differential response (Fig. 1-A) suggested a positive gradient of “IR-sensitive cells” from the stem cells up to the elongation zone. In irradiated H2B-YFP, the two times-lower density of cells within the first 300 µm of the root tip (15 *vs* 30 cells) (Fig. 1-B), indicated a loss of the progressive longitudinal and radial increase in nuclear size and number of mitotic figures in the transition zone. Epidermis and endodermis cells with high DNA content, which were located above the division zone in controls, were close to the remaining meristematic cells, which contained a lower DNA content (Fig. 1-B). Together, these findings suggest that most of the early IR-arrested cells exit the cell cycle without further division within 1 to 2 d post-IR to undergo accelerated differentiation. The range of DNA contents of seedling roots oscillates between 2 C and 4 C, respectively, in G1 and G2 diploid or G1 tetraploid cells, up to 8 C and 16 C, in endoreduplicated cells and the relative repartitioning of cells between the phases depends on the ecotype and the development stage [63], [66], [70]. Therefore, the distribution of cells between the G1 and G2 phases estimated from the CYCB1;1-GFP pattern (independent of DNA content) could not be superimposed with the DNA content estimated by H2B-YFP. Instead, the relative increase in the number of cells with a high DNA content in the root tip after IR (Fig. 1-B), consistent with cytometry data [63], [71], might indicate that endoreduplication occurred in early IR-arrested-and prematurely differentiating-cells. Protoxylem in that subzone undergoes programmed cell death during differentiation into metaxylem. If dead stele cells were occasionally observed in controls, their number clearly increased after IR (Fig. 1-B and C), therefore indicating an accelerated differentiation of protoxylem and/or their differential sensitivity to IR compared to ground tissue. Stretched and/or dead cells coincided with an auxin increase in the provascular tissue without a change in the accumulation pattern relative to vascular cells, as DR5-GFP fluorescence was continuous along the stele (Fig. 1-C) when it was restricted to the columella cells in controls. Both the premature differentiation of root cells and increased vascular cell death might trigger growth arrest due to changes in auxin homeostasis. The auxin increase was subsequent to CYCB1;1 accumulation, however, suggesting that early IR-activated cell cycle checkpoints primarily determined growth arrest and were quickly followed by changes in auxin distribution and response. Together, these data indicate that transient root growth arrest for 1 to 2 d after sublethal IR (Fig. S2) resulted from two main events: immediate cell cycle arrest in the meristem zone (1–3 h post-IR), followed by differentiation/enlargement of a majority of cells (1 d post-IR), while a subset of cells corresponding to the stem cell zone were arrested for a longer period of time before restart of growth. Consistent with recent studies of *Allium* meristematic roots cells that stop mitosis 4 to 5 h after IR and double the number of cells at G2 for approximately 20 h before they restart growth 2 to 3 d post-IR [71], these data demonstrate that the nearly immediate cell response to IR is heterogeneous and results in complex developmental patterns.

### IR promotes early defects of root stem cell maintenance that precede progressive meristem consumption and death of the *atm* mutant

**Figure 2 pone-0000430-g002:**
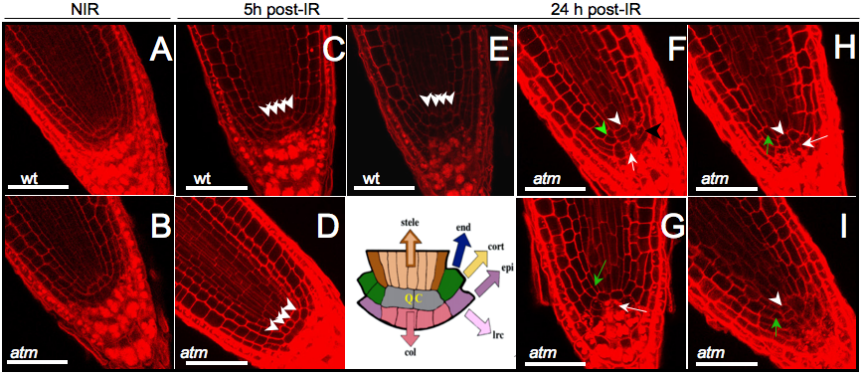
CLSM optical longitudinal sections of WT and *atm* stem cells and QC post-IR. Arrowheads point to QC cells and arrows on columella initials. The drawing shows the QC, which contains cells that rarely divide in WT surrounded by initials of stele (brown), endodermis and cortex (green), epidermis and lateral root cap (violet), and columella (pink). WT QC and initials have a normal structure after IR showing periclinally-orientated cells (A, C, E). Anticlinal division of 1 QC cell occurred 1 d post-IR in atm (white arrowhead, F–I) but not 5 h after IR (D). QC cells were either surrounded by dividing cortex and endodermis (green arrowhead) and columella (black arrowhead) initials (G) and/or differentiation of these initials (green and white arrows, respectively) in atm (F–I). Bars = 50 µm.

After root lengthening 2 to 3 d post-IR, radiosensitive *atm* mutants were completely arrested, whereas WT roots had restarted growth [46]. Longitudinal sections of fixed root meristems showed no changes in tissue organization 5 h after IR in either *atm* or WT (Fig. 2A–D). One day later, columella root cap and cortex/endodermis initial cell numbers in irradiated WT were equivalent to those in controls, showing periclinal division of initials and indicating maintenance of the QC and stem cells, except for the above reported premature differentiation of cells located far from the initials (Fig. 2-E). On the other hand, in irradiated *atm* roots early all initials had disappeared or were altered (Fig. 2 F–I) down to only two QC cells immediately surrounded by differentiated endodermis, cortex, and disorganized columella initials containing starch granules. This phenotype is similar to the loss of division capacity in columella initials that undergo differentiation after QC ablation [72], [73]. Nearly synchronous ectopic anticlinal, but no periclinal, division of irradiated *atm* QC cells, lateral root cap, and epidermis initials was also observed together with enlarged non-dividing QC cells next to the other anticlinally dividing QC cell. Wild-type plant QC cells occasionally self-renew to replenish initials displaced from their position, rendering them hard to detect. Therefore, ectopic anticlinal stem cell and QC cell division cell together with the loss of columella initials indicated an early loss of function of stem cells in irradiated *atm* and suggested a combination of stem cell-restricting (in initials) and stem cell-promoting (in 1 QC cell and initials) events. The stem cell-promoting events were remarkably similar to those induced by overexpressing and silencing genes in the canonical retinoblastoma-related (RBR) pathway [73]. The stem cell-restricting events were similar to those observed after ectopic expression of CDKF;1, which results in decreased CDKA;1 activity [74]. These observations suggest that the QC cannot fulfill positional signaling roles and control of initials fate after IR in *atm* seedlings, and that ATM likely controls decisive checkpoints for stem cell maintenance.

**Figure 3:**
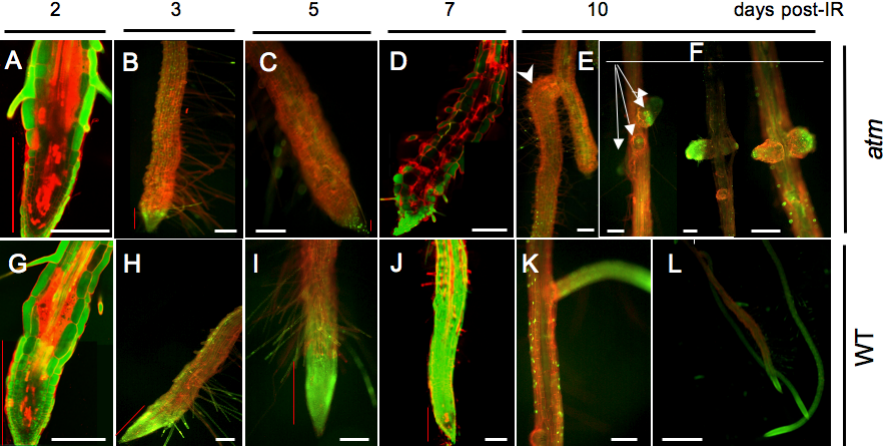
WT and *atm* root development time-course after IR. At the indicated times post-IR, seedlings were stained with PI and either FDA (green cytoplasm) or sytogreen (green nuclei). A, G, D, J are CLSM optical longitudinal sections of FDA-stained roots, and the other images are fluorescence micrographs. Arrows show abnormal (E) and incorrect positioning (F) of lateral roots in *atm*. (D) typical irradiated *atm* root, the morphology of which is similar to *short root, korrigan, shepherd, tonsoku*, and brefedin A-treated *scd1-1* mutants, propyzamide-treated WT, or *cyclin B1;1* dominant negative mutant (A) to (K) bars = 150 µm; (L) bar = 1500 µm. Red vertical bars indicate the lateral root cap zone, the size of which correlates with root meristem survival.

**Figure 4:**
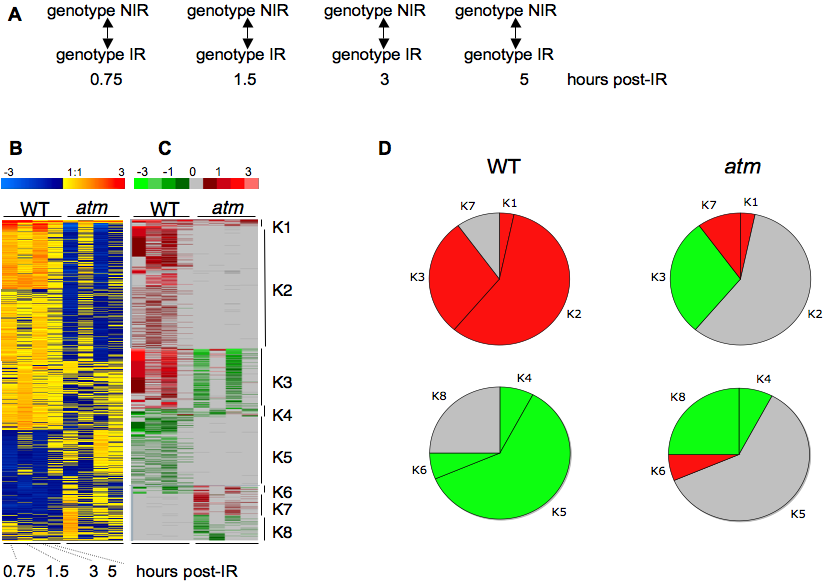
Radiomodulated genes in WT and *atm* seedlings over 5 h post-IR. There were 1713 genes with at least one statistically significant change (Bonferroni p-value≤0.05) in WT and/or *atm* seedlings. (A) : Experimental design for the time course of transcript profiling in WT and *atm* (genotype) after IR. IR, irradiated seedlings; NIR, non-irradiated seedlings. (B) : Clustering by K-means with Genesis software (all other ratios with any Bonferroni p-values). (C): Manual clustering (all ratios with Bonferroni p-value≤0.05). Ratio scale is on top of each image. Genes of clusters K1–K8 are listed in Tables S1.1 and Table S1.2. (K1–K3) : genes upregulated in WT, and either upregulated (K1), invariant (K2), or downregulated (K3) in *atm*. (K4–K6): Genes downregulated in WT and either downregulated (K4), invariant (K5), or upregulated in *atm* (K6). (K7–K8): genes invariant in WT and either upregulated (K7) or downregulated (K8) in *atm*. (D). Relative distribution of invariant (grey), up- (red), and downregulated (green) genes in WT and *atm*. All unique genes are compiled in Table S3-A. The gene clustering methodology is described in Fig. S3.

Confocal laser scanning microscopy images of root tip viability confirmed the higher density of dead protoxylem cells in the *atm* meristematic zone 2 d post-IR (Fig. 3-A). Irradiated roots were bent and enlarged, and developed root hairs in closer proximity to the tip, a hallmark of differentiation due to premature exiting of the cell cycle. Wild-type root cells maintained an ordered structure in the stem cell area, and developed lateral roots of the proper size and shape, although only a few developed close to the tip, a pattern likely linked to the above-mentioned disturbance of the auxin dose. The major feature of irradiated *atm* was the progressive death of meristematic and promeristematic cells (Fig. 4 b–d), ending with a mass of apolar cells of abnormal size and shape. This phenotype is similar to that of developmental mutants depleted in various genes such as *SHORT ROOT* or cell cycle and growth-associated genes (Fig. 3). Moreover, *atm* hardly developed lateral roots and initiated improperly located primordia that usually aborted or developed into abnormal roots (Fig. 3E). Thus, although lateral root initiation *per se* occurred in *atm*, indicating its capacity to divide for a while, the capacity of primordia to sustain both the cell division and correct polarity required for a true novel organ was lost. Such patterns are consistent with the elimination of cell cycle checkpoints and suggest that an event subsequent to division was responsible for the definitive failure of *atm* to recover from the IR in primary and secondary roots. The late *atm* phenotype might also be due to defective repair in addition to early cell cycle checkpoint defects.

### IR triggers a large wave of radiomodulated transcripts that are strongly impaired in *atm* seedlings

To describe the consequences of ATM depletion on transcriptional changes occurring during cell division arrest post-IR, genome-wide expression profiling of seedlings was performed using the Complete Arabidopsis Transcriptome MicroArray during the period covering the CYCB1;1 accumulation (see Fig. 4-A). A total of 1710 genes that had a statistically significant change in expression after IR (Bonferroni p-value≤0.05) at least once in WT or *atm* (Tables S1.1, S1.2) were distributed in K1–K8 clusters (Fig. S3-A). Gene radiomodulation in WT occurred as an early wave starting just after IR and lasting approximately 3 h with only approximately 10% of the genes still fluctuating 5 h post-IR (Fig. 4-B andC). While a subset of genes was continuously expressed over 3 or 5 h post-IR (K1), another subset followed a biphasic regulation *vs* time (K3), indicating that seedling cells still experienced differential gene cycling after IR. In addition to the phase shift in gene cycling between control and irradiated seedlings over time, the oscillation of a subset of transcripts might also indicate differential cell reactivity to IR within seedlings (*e.g*., dividing *vs* differentiating, cotyledon *vs* root) and/or differential transcript stability after IR. The transcriptional control of genes was lost in *atm* (Fig. 4-D). The expression of only 35 genes was upregulated in *atm* but the expression was still significantly lower than that in WT and/or delayed (cluster K1), while 632 genes were invariant (cluster K2) and 314 were inversely regulated (cluster K3) in *atm*. Oscillation of a subset of genes was also observed in *atm*, suggesting that transcript level oscillations *per se* were independent of genotype. Whereas K1 and K2 genes exclusively required ATM to be upregulated for 5 h after IR, the inverse regulation of K3 genes and a subset of 110 genes exclusively upregulated in *atm* (K7) suggested that other factor(s) acting concomitantly with ATM tend to repress or induce the expression of gene subsets after IR. These observations also applied to downregulated genes (clusters K4–K6, and K8). Together, the data indicated that (i) the main effect of IR was to immediately trigger the upregulation of a large number of genes concomitantly with CYCB1.1 accumulation and division delay, (ii) *atm* mutation resulted in attenuated, canceled, or reversed IR-regulation of most transcripts, (iii) biphasic oscillation of a subset of up- or down regulated transcripts concomitant with the continuous expression of other genes, independently of *atm* mutation, suggesting that seedling tissues have a differential response to IR.

Real time quantitative PCR (rt-qPCR) to validate array data was performed for 51 randomly chosen genes, invariant, up-, or downregulated (Table S1-primers). The highest ratios usually gave higher-fold changes by rt-qPCR than by ratio-dependent calculations, and lower ratios around 1, theoretically indicating a 2-fold change, gave values up to 8 times higher by rt-qPCR for some genes (Fig. S4-A). The high stringency of the statistical treatment according to Bonferroni criteria (Bonferroni p-values≤0.05) was confirmed for genes that were barely detected, such as PARP or DNA polymerase ε, or not detected (*ku70, lig4, brca2*) by microarrays. These genes were upregulated 2 to 4-fold, as detected by rt-qPCR, due to the higher sensitivity of the method. A comparison of WT and *atm* samples also validated the microarray data (Fig. S4-B), demonstrating that approximately 90% of the transcript level increase was lost in the mutants and that various genes had different oscillation patterns after IR. The levels of transcript variation were close to those reported in studies on DNA repair gene changes in yeast and *Arabidopsis* mutants [57], [75], but higher than those in human cells, whose levels change around 1.2 to 1.5-fold [59]. Because the statistical treatment provided highly confident results even for minimal threshold ratio-values of 0.65±0.1 (theoretical modulation of 1.5 fold), the direction of gene regulation rather than the ratio values was considered for further analysis.

### IR triggers early transcriptome changes in *Arabidopsis* roots, which are mainly ATM-dependent and weakly ATR-dependent

**Figure 5:**
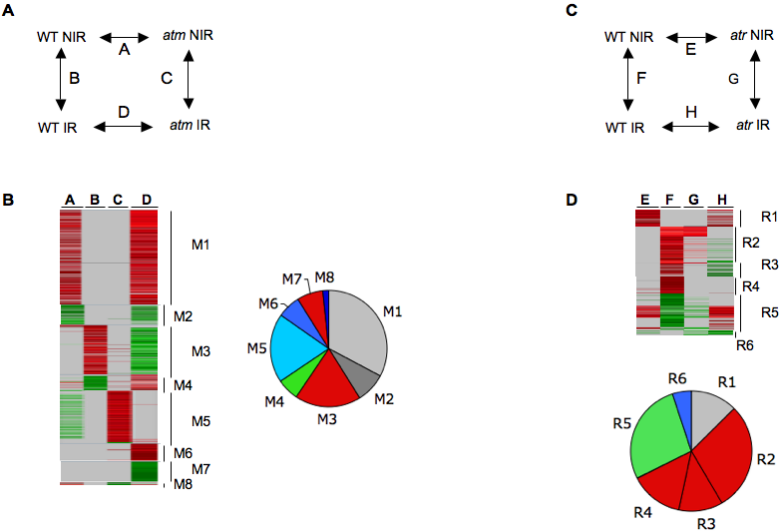
Radiomodulated genes in WT, *atm,* and *atr* roots 1 h after IR. (A and C) Experimental design of WT and *atm* (A), or *atr* (D) root transcript profiling. Combinations of samples A–D are indicated on top of columns in Tables S2, S2.2, and S2.3. (B) Clustering of 1352 unique genes from two independent biologic samples containing approximately 200 (expt.1) and 100 (expt.2) roots, whose average sizes was 7.1+/−0.8 and 7.3+/−0.9 mm for *atm* and WT, respectively. (D) Clustering of 475 unique genes from one experiment done with 100 WT or *atr* roots. Clusters M and R unique genes are listed in Table S3-A. The gene clustering methodology is described in Fig. S3.

Given the emergence of gene groups with complex transcription profiles in seedlings, the differential sensitivity to IR and/or asynchrony of the response of roots and cotyledons-shoot apical meristems might be randomized within the seedlings examined. Therefore, we looked for early radiomodulation of genes in WT and *atm* with roots that provided a tissue homogeneous enough to obtain more clear-cut transcriptional responses. The experimental design shown in Fig. 5-A provided relative gene expression in both genetic backgrounds before and after IR, as well as autovalidation of the results (Fig. S3-B and C). From two independent experiments performed with 200 and 100 roots, 664 and 1110 genes, respectively, had at least one significant change in expression under one of the four conditions (Tables S2.1 and S2.2), resulting in a set of 1457 genes (Table S3-A) and 317 genes that were regulated in both experiments. This showed that increasing tissue homogeneity and lowering the root population size increased the detection sensitivity and/or indirectly confirmed cycling of gene expression after IR. Genes showing the most technically relevant changes were located in clusters M1 to M4 (Fig. 5-B). Clusters M1 and M2 displayed a high number of genes that were not radiomodulated, but constitutively over-and under-expressed in *atm*, indicating that *atm* has higher transcriptional activity than WT. Clusters M3 and M4 included genes without differential expression between WT and *atm* before IR and displayed 251 up- and 83 downregulated genes, respectively, in WT, and invariant or severely attenuated gene expression levels in *atm*. Genes that were radiomodulated in only one experiment and/or in only one sample instead of two (clusters M5–M8) had a more stochastic expression that was likely related to the gene oscillations observed in seedlings. For example, the largest cluster M5 cluster mainly overlapped with the cluster K3 (Fig. S5-A), therefore confirming the misregulation of genes in irradiated *atm*. M7 genes, which were upregulated in WT and invariant in *atm*, behaved similarly to cluster M3 genes when the ratios were examined (Table S2.2). Therefore, they were associated with cluster M3 for further analysis (M3–M7, Table S3-A). Together, the root experiments confirmed all trends of gene expression observed in the seedling experiments and extended and helped to distinguish the set of genes whose radiomodulation was strictly ATM-dependent after IR from those that were cycling abnormally.

Because the checkpoint kinase ATR controls G2 arrest in *Arabidopsis* [47], a root experiment was performed with WT and an *atr* mutant (Table S2.3, Fig. 5-C and D). A small set of IR-invariant genes (R1) was expressed at higher levels in *atr* before IR, indicating a slightly higher transcriptional activity in *atr* but considerably lower than that in cluster M1 (Fig. 5-D). The key feature was the occurrence of two large gene sets of either strongly upregulated (cluster R2) or downregulated (cluster R5) genes that were similarly radiomodulated in WT and *atr*. Cluster R2 included cluster K1-M3 genes (Table S3-A), but a subset of them had slightly attenuated expression in *atr*. Other cluster R2 genes did not show a statistically relevant change in irradiated *atr* (Table S3-B). They behaved like R3 and R4 genes, which were in lower ratios in WT. As they had ratio-values close to the minimal threshold value for detection, however, we concluded that their IR-induced expression was attenuated rather than strictly invariant in *atr*. In addition, with a few cluster R1 genes, and no clusters showing the diversity of regulation patterns that occurred in the *atm* experiments, these data showed a weak effect of ATR depletion in the early transcription response to IR compared to ATM.

**Figure 6:**
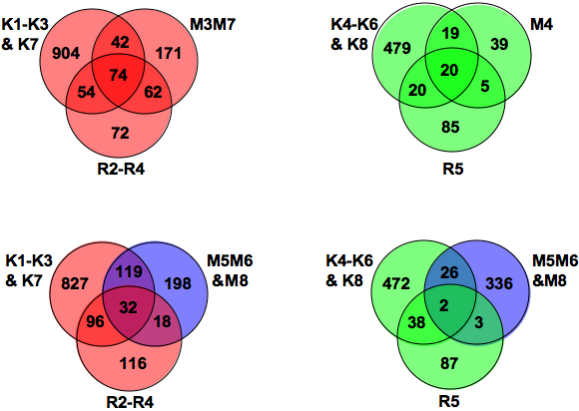
Distribution of radiomodulated genes in roots and seedlings. Venn diagrams show the extent of overlap between roots [3 experiments (M and R clusters)] and seedlings [8 experiments (K) clusters)]. (Red and green) : Up -and downregulated genes. (Blue): Genes with stochastic expression in *atm* roots. Minimal cores of genes for which expression was lost in *atm* (diagrams on top) were extracted in Table S3-B as follows: Group A included 74 (up) and 20 (down) genes regulated in all experiments; Group B (42 and 19 genes, respectively) and Group C (54 and 20 genes, respectively) included genes regulated at least once in seedlings and at least once in roots; and Group D included 62 (up) and 5 (down) genes regulated in root experiments only.

The extent of overlap between genes IR-regulated in roots and seedlings as well as the dependence on the number of experiments to get a complete IR transcriptome is summarized in Fig. 6. This representation does not highlight all reproducibility levels of gene expression, as genes within root and seedling clusters that did not overlap were either more than once or highly expressed. Therefore, the transcriptome content was further analysed from data compiled in Table S3-A. Cell cycle characteristics and distribution in functional classes showed enhanced S- phase gene regulation and reduced M-phase gene regulation in irradiated roots (Fig. S6-C), consistent with the tissue distribution of proliferation (Fig. S6-B). This coincided with an enrichment of metabolism genes in the aerial part of the seedlings (Fig. S6-D–E), and was consistent with the low division competence of cotyledons [76].

### ATM mediates IR- induced expression of cell cycle G2/M and G1/S checkpoints

Cell cycle indicators of proliferation arrest included downregulated S-phase CYCA3;2, and mitotic and G2/M-phases activators, such as cyclins B1;3, B1;4 B2;2, and A1;1, CDKB2;1; an APC activator, AtCDC20.2; the kinesin-7 CENPE, the kinases AURORA1 and 2 and MAP3K14, and KNOLLE, which are hallmarks of highly dividing plant organs [77]. Others encoded orthologs of spindle-chromosome components that link the regulation of their attachment to mitotic checkpoint signaling in vertebrate cell division, such as AtEBC1, TPX2-like, a regulator of RanGTP gradient (RanBP1), and the transient centromeric checkpoints AtBUB1 and AtBUB3, providing further evidence of arrest outside of M-phase. Cell cycle inhibitors, such as the known AtCcs52A1 [78], usually expressed from late M until lateS-early G2 phases, were upregulated, as well as novel genes such as the orthologues of NUP98, a mouse temporal regulator of APC that maintains euploidy by preventing premature separation of sister chromatids, and the human mitotic checkpoint protein CHFR, a non-canonical ubiquitin ligase that delays chromosome condensation by keeping AURORA-A and-B inactive, but also inhibits the entry of CYCB1 in the nucleus, and therefore delays mitotic progression [79]. In contrast to all other B-type cyclins that were downregulated, *CYCB1;1* was quickly induced slightly before protein accumulation (up to 1.5 h post-IR, Table S1.1), and was later invariant, whereas CYCB1;1-GFP protein accumulated for several hours (Fig. 1), likely indicating transcriptional and posttranslational regulation. Indeed, *CYCB1;1* is the only B-type cyclin that, although upregulated during cell cycle re-entry, does not show significant subsequent changes during cell cycle progression [80], [81]. In addition, ectopic expression of CYCB1;1 under control of the At*CDKA* promoter, a G1/S-active CDK expressed uniformly throughout the cell cycle, markedly accelerated plant growth without altering development, raising the possibility of an unknown CYCB1;1 function in the G1 phase [82]. This strongly suggested that contrary to other B-type cyclins, CYCB1;1 is positively regulated at S phase after IR, as its activator of quantitative expression TCP20 [83] was also upregulated by IR. Together, with the plant CDK inhibitor KRP6 expressed at the M/G1 boundary [81], two novel putative G1/S regulators, orthologues of hGSPT1 (G1 to S phase transition protein 1), a cell cycle regulator that interacts with RNAseL at translation termination [84], and of hSYF2, a splicing factor of the Grap2 CYCD-interacting protein family AtGCIPp29 that inhibits activity of the S-phase transcription factor (TF) hE2F1 [85], indicated IR-induced activation of the G1/S checkpoint. KRP6 is highly expressed in the roots, however, in both mitotically dividing and endoreduplicating cells and interacts with D-type cyclins [86]. It binds more strongly to active CYCD2/CDKA;1 and CYCD2/CDKB2;1 complexes than to their monomer components [87]. *CDKA;1* and *CDKB2;1* are expressed throughout the cell cycle, and from early G2 to M, and CYD3;1 (not CYCD2) interacts with CDKA to dominantly drive G1/S transition [77]. After IR, the expression of *CDKA* and *CYCDs* was unchanged, while that of *CDKB2;1* decreased, suggesting that KRP6 inhibits cell cycle progression at the G1/S and early G2/M transitions through different mechanisms to block division after strong DNA damage or in natural situations of increasing DSBs, like in meiosis [88]. Similarly IR-induced upregulation of the negative regulators of CDKA;1 activity, WEE1 and PAS2, expressed at S-phase likely reinforce division arrest. Indeed, the growth of PAS2^OE^ or WEE1^OE^ plants is strongly inhibited as cell division is delayed from G2/M to early M, resulting in enlarged, highly vacuolated root cells [89], [90]. In this context, upregulation of ATGR1, whose elevated intracellular levels are associated with changes between the G1/S and M phases of the cell cycle that trigger somatic cells to enter the endoreduplication cycle, and/or cell enlargement [91], as AtCcs52A1 and non degradable CYCB1;1 do [92], [93], might be an important S-phase regulator after DNA damage. Altogether, the repression of M and S phase activators and the increasing levels of KRP6 and CYCB1 proteins suggest that cells likely arrested both at S and G2 through activation of a complex network, and that transition from proliferation to endoreduplication might have occurred in irradiated seedlings. The finding that these genes were essentially invariant in *atm* after IR indicates that ATM-DNA damage-mediated cell cycle checkpoints influence the capacity of mutants to survive.

### ATM controls IR-induced upregulation of Arabidopsis genes involved in DNA replication, repair, and recombination and chromatin metabolism

DNA synthesis-associated genes were downregulated, including a DNA replication factor RPA, 12 histones (including several H4-type histones [81]), and AtMCM4, which is a target of the DNA replication block checkpoint system in human cells [94]. IR-upregulated genes (Table S3-A) included major mediators of DNA repair by HR such as BRCA2, BRCA1, RAD51, AHP2, MND1, and RAD54; the homologues of hFANCD2 [95] and hFANCJ/BACH1 helicase; the ssDNA binding protein RPA1; and RAD17. Novel genes might identify putative proteins related to cell cycle checkpoints or DNA repair, such as BRCT-or FHA-containing genes [32], or a predicted DNA topoisomerase-related gene that encodes a protein structurally close to TONSOKU-ASSOCIATED 1 (TSA-1-like), which is involved in the DNA damage response, epigenetic silencing, and proper cell arrangement in meristems [96]. The novel genes also included AtRAD21.1, AtSHUGOSHIN1*-like*, a sensor of tension between sister chromatids, the condensin/cohesin *AtDELANGIN-like*, and two proteins with similarities with non-structural maintenance of chromosome subunits of the HR-SMC5-6 complex (SpNse1 and SpNse4/ScRad62) that collaborate in repairing DNA damage and maintaining chromosome integrity during replication [97]–[101]. The IR-induced upregulation of TK, 3 subunits of ribonucleotide reductase, the POLε catalytic subunit AtPOL2, 2 subunits of the processive DNA POLδ, and AtPOLK involved in translesion synthesis [102], [103], confirmed the mobilization of replicational repair proteins, which often interact with RAD17 to stimulate DNA repair activities in human cells [104]. Moreover, the upregulation of the DNA replication licensing factor MCM3, which interacts with RAD51 and is directly involved in ATM/ATR checkpoints in mammals [32], [105], [106], might indicate the activation of cell cycle G1 and/or S checkpoints. In addition, the upregulation of PARP1 and PARG, involved in the control of protein ADP-ribosylation at sites of damaged DNA and of the balance between NHEJ and HR in mammals [107], [108], AtXRCC1 putatively involved in SSB repair, or AtCEN2, a modulator of HR/NER [109], indicates the occurrence of IR-induced DNA lesions other than DSBs (SSBs and/or multiple-base damage [4]). Remarkably, the expression of the NHEJ components At*ku70* and At*ligIV* increased 2 to 4-fold in WT, which was barely detectable in microarray analysis and similar to that induced after a 10-fold lower IR dose [110], suggesting poor IR-regulation compared to HR genes in WT. As their expression is only delayed in *atm* [46], NHEJ is likely functional in the mutant. The *tebichi-1* mutant, which is defective in a DNA POL-helicase close to human translesion synthesis POLs, constitutively expressed HR and S-G2 genes (Table S3-C). This shows that plants defective in non-HR functions shift towards expression of HR functions, as shown by the very high induction of BRCA1 and RAD51 in bleomycin-treated At*ku80* mutants [55]. Finally, the upregulation of the DNA methylase DRM1 and the helicase hNDHII-like that colocalizes at DNA damage-induced sites of arrest transcription and replication [111] suggests that transcription domains were reprogrammed.

The concomitant upregulation of several genes encoding chromatin-associated proteins indicated a persistent IR-induced alteration of chromatin conformation, a hallmark of cancer or irradiated human cells [43]. They encoded high mobility group proteins (HMGs), several uncharacterized proteins that harbor chromatin-specific modules (PWWP, SWIB, AGENET, BAH, SET), and notably, the histone methylase AtASHH and the GCN5 acetylase AtNSI, which modify histones and virus coat proteins [112]. Radiomodulation of several regulators of chromatin condensation (RCC1) and TOM proteins that bind to viral replication proteins [113], telomere repeat factors-like AtTRFL10 and 3, and a GANP family member, which facilitates the nuclear localization of hMCM3, illustrates the broad range of modifications of DNA, histones, and non-histone proteins that occur after DNA damage. Concomitantly, HMGB6 and 3 histone deacetylases (HDT1/2/4) that repress transcription through interaction with TFs [114], and a mutator-like transposase and 2 transposons were downregulated, suggesting increased decondensation sectors of chromatin and changes in methylation patterns leading to transcription silencing [115]. ARP4, the mediator of NuA4 histone acetyl transferase (HAT) remodeling complex that is recruited at DSB sites to remodel and open chromatin for DNA repair [116], and HAG4, a putative homologue of hTip60 acetylase, were oppositely regulated but not invariant in *atm*, suggesting indirect ATM-regulation. Arabidopsis mutants impaired in chromatin-associated factor 1 (CAF-1) constitutively express 20 IR-induced upregulated genes, including CYCB1;1, and mainly DNA HR genes (Table S3-C). CAF-1 deposits a specific histone variant in nucleosomes that is active primarily at the time of DNA synthesis (replicative or repair), suggesting that IR might cause similar defects in DNA and chromatin structure. E2Fa-DPa^OE^ seedlings constitutively expressed 32 class 1 ATM-and IR-induced upregulated genes (Table S3-D). Twenty of these genes encoded major DNA replication and HR repair proteins, that, except for a few, harbor an E2F consensus motif or have S-phase expression [117]. The negative IR-induced regulation of DNA synthesis and S-phase genes (CYCA3;2, 8 histones, MCM4, RPA, HMGB6, BARD1) shows that DNA damage checkpoints selectively counteract S-phase cell cycle checkpoints, resulting in dual regulation of the S-phase TF E2Fa/DPA activity. Together, these data indicate that the large range of DNA lesions leads to a strong reorientation of chromatin metabolism. The incorrect expression of class 1 genes in *atm* and weakly *atr* suggests that DNA damage is signaled by the kinases.

### ATM controls IR- induced upregulation of genes involved in cell reprogramming

**Figure 7:**
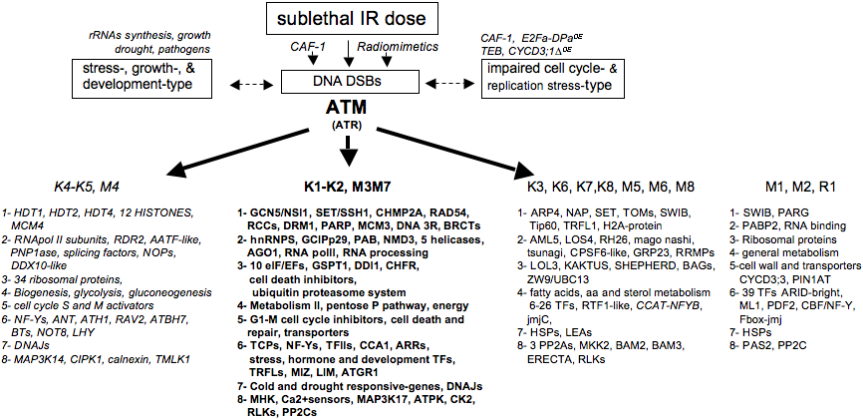
Hallmark genes in IR-and ATM-mediated transcript profiling. IR induces DNA lesions resulting in different types of chromatin alterations that also occur through genetic or physiologic disturbance. Examples of downregulated (italics) and upregulated genes (bold) in each functional class (1–8) are shown. Examples of misregulated (up or down) genes (standard font) and constitutively deregulated genes (no arrow) in *atm* or *atr*. Genes are extracted from Table S3-A.

In the context of severe cell cycle delay accompanied by strong DNA and chromatin metabolism, the concomitant up- and downregulation of genes within each class illustrates the strong reprogramming of cellular functions. Genes involved in translation, protein turnover and cellular trafficking, transcription, and signaling demonstrate non-random changes (Fig. 7). Genes with decreased expression were mainly involved in basic cellular activities and the associated regulatory infrastructure, such as RNA processing and splicing (fibrillarin, PNP1ase), translation (eIFs, NOP56, RPSOB), morphogenesis (expansins, nodulins, cell wall proteins), or essential metabolism genes, e.g., during nutrient-induced reprogramming [118]. Similarly, downregulated genes encoding TFs, a PP2C gene that is quickly downregulated by cold stress, together with calnexin and a RhoGAP, which are pivotal switches acting in Ca^2+^ signaling and the cytoskeleton during plant tip growth [119], [120], indicate general growth arrest. Concomitantly, upregulated genes in class 2 included several genes that have central roles in RNA metabolism (NMD3, ScENP1-like, AtPAB8, EMBs), DNA repair, telomere biogenesis, cell signaling, and gene expression, such as heterogeneous nuclear ribonucleoprotein particles and RNA helicases (hDDX8-like, AtLOS4). Accordingly, regulation of the translation apparatus and turnover of proteins such as FKBPs, eIFs, proteases, and CHFR; and regulatory components of the proteasome and the ubiquitylation machinery (e.g., RCE2, ubiquitin ligases BRH1, KAKTUS, HAKAI), 20 F-box proteins (e.g., SKIP2, FBX13 and 3, FLB6, KELCH-F-box) indicated superimposed cell cycle and hormonal-dependent responses. We identified ScDDI1-like, a UbL-UbA protein involved in the MEC1/ATR-mediated turnover of an F-box protein [121], hPSO4, or IBR-RINGs, indicating a regulation of nucleic acid metabolism. Broad-spectrum TFs (TFIIs, CBF/NF-Ys, TCPs/PCFs, NOTs) as well as stress-specific TFs were identified accordingly (class 6). Notably, a repressor of proliferation TCP4, and the activators AtTCP-20/PCF1 and OsPCF2-like, which drive quantitative expression of CYCB1;1 and ribosomal proteins respectively [83], [122], and of DNA synthesis-related genes, likely identify part of TFs governing IR-specific S-phase and DNA replication-associated transcription. Furthermore, upregulation of AtPurα, which physically interacts with AtTCP20 and AtE2F [123] and controls the expression of G1 and S-phase genes (translation apparatus, RNR, TK), indicated the IR-induced regulation of active E2F titration. WRKYs and AP2s, which are the most abundant pathogen- and cold regulated factors under the control of ICE1 [119], ICE1, ZnFs, and BTs, were the major groups followed by developmental factors (homeobox, NAM, SCARECROW-like, TOPLESS-RELATED, HANABA TARANU-RELATED, MIZU-KUSSEI1, AGAMOUS-LIKE18), and hormone-responsive factors (EREB, ARF2, BZR3, CRF6, ARGOS, AXR2, MASSUGU2) [124]. Uncharacterized TFs, such as the single predicted TFs with a LIM (OsSF3-like) or a MIZ domain, identified new factors of the IR-induced response [125]. Dehydration- and disease-responsive genes were the most abundantly modulated stress induced-genes (class 7), followed by high salt, wound, cold, or senescence-associated genes. Notably, more than 10 HSP/DNAJs co-chaperones might indicate a strong requirement of cell repair as well as important TF translocators [126]. Other cell repair genes and attenuators of cell death (AtBI1, BAG3, BAG7, autophagy8h, NDP-kinase, NUDT7) were induced together with cell death genes (ACD2, RCD1, VAD1, MCP1b) and might signify strong cellular damage and links between hormone and stress signalling after IR [127]. The most abundant group in class 8 encoded Ca^2+^ sensors (7 Ca^2+^-/CAM-binding proteins, CAM9, CDPK19, CIPK11, and a new CIPK1-IP (ECT2) that relays the cytosolic Ca2+ signals to the nucleus. Together with the induction of AOS-transducers (ORG1, MAP3Ks, OXI1), and hormone- mediators (RLKs, RCN1, RACK1), growth-related kinases (AtS6k2, IPK2a, CK2B3, 4 PPC2) [128], confirmed the large reprogramming, consistent with the nature of regulated TFs, and signaling or stress-responsive genes. Several genes in both classes were constitutively expressed in mutants, suggesting that IR as well as chromatin defects regulate part of the cell cycle progression through calcium signaling (Table S3-C) [129]. Together, the data indicated that genes in all classes are required for essential functions characterized as cell development under general stress.

### Specific gene expression in ATM-depleted mutants reveals constitutive defects

Clusters K7 and K8 contained genes invariant in WT that were modulated for usually less than 1 h post-IR in *atm* (Table S1.1). Within hundreds of upregulated genes in cluster K7, we distinguished two main sets of genes. One includes genes involved in transcription and development such as the orthologue of BLOCK in PROLIFERATION 1 (hBOP1), SMT1, ATHB5, TCP1, AtCUL1, GRP23, or ankyrin repeat proteins, all implicated in the evolution of key morphologic traits and functions [122], [130]. The other group comprised many oxidative stress-related proteins (AtPP2-A5, OZI1, LTV1, AtGPX6, AtMYBL2). Remarkably, the upregulation of SpCDC5-like and SpPRP19/hPSO4-like [131], both essential proteins for interstrand crosslink processing in a specific error-prone recombinational repair pathway [132]. Early downregulated K8 genes included uncharacterized genes such as a DNA storekeeper protein, AtTRFL1, the acetyltransferase AtHAG4/hTIP60, an upstream stimulator of ATM activity. Notably, they included SCL genes involved in specification and maintenance of the QC stem cells, ARRs, pinoid-BP, and PP2A phosphatases (RCN1, PP2AA2), and TANGLED1 homologue ATN, which have a cardinal role in hormone-mediated growth regulation as well as in the control of cell shape and plant morphology [124], [133]. Clusters M1 and M2 included genes with constitutive expression defects in *atm*. M1 included uncharacterized genes such as NOL1-PCNA/NOP2/sun family protein, and a histone-like TF (CBF/NF-Y), similar to hNF-YB that pre-sets the promoter architecture for access to other regulatory proteins, and often associates with E2F to regulate transcription during the cell cycle [134]. M2 genes displayed several nodulins, hormone responsive/regulated and permease (AtPUP4, EXP1, IAAs, CYCD3;3), PAS2, and PIN1AT, the depletion of which induces premature mitotic entry and mitotic arrest in yeast [135]. Another M2 hallmark was a F-box-TF jmjC-like, which is close to new human chromatin modifiers and/or to a transcriptional repressor of human CYCD1 [136], [137]. Clusters K3 and M5, which largely overlap (Fig. S5-A), included chromatin proteins (ARP4, SWIB, SET, nucleosome assembly protein), but neither DNA 3R genes nor cell cycle regulators, and were strongly enriched in RNA, protein, and primary and secondary metabolism functions (Fig. S5-B). This shows a bias towards functions required for cell and organ growth and polarity (HYD1, STE1, FROSTBITE1, KOR, HSC70s, KAK, ZW19, MERI-5, RCD1). Altogether these expression patterns identify additional key genes involved in the developmental and signaling pathways that were revealed by increasing the number of experiments and comparing the status of transcription before and after IR in WT and mutants. Furthermore, these patterns indicate that ATM does not directly control other signaling pathways that are required for a correct transcriptional response to IR.

### ATR weakly controls early IR-induced ATM-dependent gene expression

Cluster R2 displayed 139 highly upregulated genes in WT and *atr* that mainly overlap with ATM-regulated clusters M3-M7 (Fig. 5-E). Twenty of these genes had attenuated expression with high statistical significance, therefore exhibiting a pattern similar to that of clusters R3 and R4 and suggesting that the maximal level of gene expression reached in WT somehow requires the combined action of both kinases in the early response to IR. Hallmark genes with such a pattern (”LOW” in Table S3-B) included RAD51, RAD21, TRFL10, NDHII, FHA-and BRCT-proteins, NRAMP3, ARAC7, USO1, AtGR1, AN1-like, and bHLH109 TFs, CIP7, and 4 proteins without domain features. There were 18 other genes with lower ratios in WT (R2-R4, « low »in Table S3-B) than in clusters M3–M7, and 11 genes that overlapped (Table S3-A). These genes encode dehydrins, LEA proteins, the TF IMB1 (seed imbibition protein 1) whose mutation causes upregulation in transposons and transposases [138], and a RNA helicase (EMB3011), suggesting a bias towards dessication-associated chromatin changes. In addition, they include sensors of topologic changes of chromatin (TSA1-like, SGO1-like, DELANGIN, PARP1), and regulators of chromatin structure (AtASSH1, ARP4, hCHMP2A-like), and notably an ARID-BRIGHT protein (Cluster R1). Finally, while the expression of CYCB1;1 and KRP6 was hardly affected in *atr*, the impaired upregulation of the G1/S factors (AtGCIPp29, GSPT1, GANP), and downregulation of APC8 (cluster R6) indicated a slight effect of ATR on cell cycle-related gene transcription at G1/S and G2/M, consistent with a slightly altered cell-cycle arrest after IR in *atr* [47]. These data indicate that ATR slightly complements ATM in the transcriptional response to IR.

## Discussion

Genome-wide transcript profiling coupled with analysis of the developmental features of *atm* and WT might help to reveal cell functions and networks that are specific or critical to plant fate after exposure to a sublethal dose of IR. As shown by CYCB1;1 protein changes in WT, most meristematic cells with an early accumulation of CYCB1;1 lose the protein 24 h post-IR and undergo accelerated differentiation, whereas in a subset of cells located around the QC and initials, division remains blocked for another couple of days prior to starting again. This results in transient loss of the root transition zone and an auxin increase in the vascular system. Consistent with organ growth scenarios [139], this non-linear pattern, in contrast to the progressive pattern of cell differentiation, suggests that titration of active regulators along the root, which are normally required far from the stem cell area to determine cell, tissue, and organ fate, might reach local critical thresholds that determine either sustained cell division arrest, exit towards non-canonical differentiation, or death. *Atm* seedlings showed an early ectopic division of the QC and initials before experiencing consumption of the meristem and massive cell swelling up to the very root tip. The concomitant transcriptional burst of several hundred genes lasted for approximately 3 h following sublethal IR in WT, and is essentially IR-ATM-mediated and weakly IR-ATR-mediated. This might be associated with the mild IR-sensitivity of *atr* plants and to its weaker but significant contribution to the occurrence of chromatin γ-H2AX foci compared to *atm* (1.2 for *atr vs* 10.5 per cell for *atm*) [47], [48], [140]. Both kinases contributed to the number of WT foci (14 per cell). Experiments performed with alleles of the WS ecotype of both mutants, at only one time-point post-IR and with a different microarray procedure, indicated the same transcriptional trend at least for the most upregulated transcript, AtBRCA1. AtATM and AtATR have close structural similarities to their human counterparts [47], [141], and are expected to fulfil similar activities as far as functions are conserved among species.

Gene expression depends on transcriptional activity and on a variety of posttranscriptional events, including the initiation of mRNA translation and RNA degradation. Indeed, comparing microarray analyses of total and polysome-bound RNAs showed that whereas IR does modify gene transcription, it affects substantially more genes at the level of translation in human astrocytes [60]. In that study, there were few, if any, genes affected at both the transcriptional and translational levels, indicating that the cells have enough pre-existing transcripts before IR and that such controls balance the level of active factors. A larger overlap between total and polysomal RNAs might have occurred in astrocytes, because the comparison was performed with ratio values greater than 1, a method that eliminated at least 90% of UV- and IR-responsive genes in human cells, whereas changes were linearly correlated to Northern blot intensities [59], [142]. Such posttranslational control occurs in Arabidopsis cell cultures after sucrose starvation, resulting in a higher overlap between total and polysomal- regulated RNAs [143]. This shows that transcript profiles highly depend on the turnover rate of mRNA and on the cycling and differentiation characteristics of cell lines [60], [144], as well as on the underlying pathways involved before and after stress. The mRNAs whose polysome association was modified after IR in human cells correlated with changes in the level of the corresponding proteins [60]. They were not a random collection but belonged to functional pathways such as cell cycle; cell death; and DNA replication, recombination, and repair. Our data showed similar components at the transcriptional level, consistent with conserved functions between plants and other eukaryotes. Indeed, slowing down transcription, translation, DNA synthesis, division competence, and biogenesis of cell compounds was concomitantly associated with increased expression of DNA replication, recombination, and repair; cell cycle inhibitors; regulators of RNA and translation; development and hormone pathways; and stress metabolism or effectors and attenuators of cell death. Numerous functional studies in human cells and yeast have established the physical and biochemical interactions between ATM and its targets, therefore the cellular functions and pathways ATM controls are well established [20]. If we consider gene homologies between plants and that mRNA regulation in Arabidopsis reflects involvement of the corresponding protein, our data provide important information for analyzing ATM/ATR-mediated IR-induced transcription patterns in plants. For example, the MCM helicase complex subunits are regulated to control replication and HR repair in human cells [105]. Human MCM4 is strongly inactivated by consecutive phosphorylation involving DNA damage/ATR-CHK1 and cell cycle/CDK2 kinases after replication arrest [94], and theAtMCM4 transcript is downregulated. Similarly, hATM phosphorylates MCM3 after IR [106], and the AtMCM3 transcript is upregulated. Furthermore, several AtATM-mediated IR-upregulated genes encoded orthologues of human HR proteins that are localized together and/or have restricted interactions with ATM in DSBs flanking chromatin (BRCA1) or with ATR in ssDNA microcompartments of S/G2 chromatin (RAD51, BRCA1, RPA, RAD17, FANCD2, BRCA2), or with the MCM complex when replication follows HR [20], [39], [105], [106]. Altogether, such data strongly indicate that plant total and polysomal transcript profiling will help to identify numerous regulators of the DNA damage response.

### IR resets nuclear shuttling of chromatin modifiers and TFs and mediators

In yeast, human cells, and Arabidopsis, the developmental and environmental signals are detected by signalling molecules, transcriptional activators and repressors that recruit HATs and histone deacetylases, respectively. Changes in acetylation and methylation of histones, promoters, and TF regulators lead to transcriptional activation or repression by nuclear factors (NF-Y/AT/E2, E2Fs) during cell life [126], [134], [145], [146]. In human cells, DNA damage involves direct ATM-mediated phosphorylation of TFs and/or their regulators (p53, NF-*k*B, SP1-related retinoblastoma control proteins, STAT1, E2Fs) [24], [147]–[151] as well as repressor complexes such as HDACs-RB-PP1 [152]. In this network, RB has a central role as it orchestrates proliferation, apoptosis, cell cycle exit, and differentiation through interaction with TFs, TF regulators, and chromatin modifiers in concert with its multiple regulations by kinases (CDK) and acetylases (CBP/GCN5) [153]. In Arabidopsis, TOPLESS, ABI-like, and AtARP4 illustrate the interplay of chromatin modifications and TF shuttling [154]–[156]. After IR (Fig. 7), downregulated histone deacetylases and upregulated GCN5 acetylase (HAT) and histone and DNA methylases likely indicate chromatin decondensation for loading of appropriate factors, driving new transcription patterns, and cell fate [136], [155]. Therefore, the function of IR-regulated TFs, either proliferative or devoted to development, hormone-mediated, cold and drought stress, and cell death [118], [119], [157], as well as translocator DNAJs [126], likely indicates the extent of cell reprogramming and of nuclear shuttling after IR. This holds true for loading of DNA repair proteins that require specific chromatin modifiers in other eukaryotes [118], [119], [157], therefore indicating that class 1 genes illustrate the extent of DNA lesions and chromatin changes triggered by IR. Together with IR-regulation of mRNA functions (NMD3, AGO1), passive epigenetic changes (replication dependent) might also be reset to an active IR-mediated status, as cytosine and histone methylases (DRM1, SET) and the histone deacetylase HDT1, which functions in rRNA gene silencing, were regulated [158]. Therefore, misregulated pre-existing chromatin modifiers and cofactors of TFs in irradiated *atm* likely contributed to its failure to modulate transcript levels after IR.

Both IR-treated and E2Fa-DPa^OE^ plants and to a lesser extent CAF-1 depleted plants, positively expressed ATM phosphorylation substrates, which promote the S-phase checkpoint in irradiated human cells. It is now established in human cells that deregulation of E2F1 by RB inactivation or ectopic expression constitutes an oncogenic stress that induces the accumulation of DSBs, elevates the expression of genes involved in the response to genotoxic stress, and triggers apoptosis [159]–[161]. Although E2F1 location to MRN/γH2AX foci can be independent of ATM, ATM is required to mediate the response to DSBs [159]. Constitutively changing the chromatin assembly rate (CAF-1) also triggers DSBs or sensitizes the cells to an increase in DSBs and results in genetic instability in plants [162], yeast [163], and human cells [164]. This might explain the similar transcription patterns observed after IR and in mutants with a deregulated DNA synthesis rate and S-phase regulation of transcription regarding DNA repair functions. In the absence of clear transcriptional regulation of E2Fs, DPs, and RB transcripts in our different samples (only DPa reached a ratio of 0.5 in early irradiated ATM, data not shown), we hypothesize that translational and/or posttranslational regulation of the pre-existing amounts of transcripts of the RB pathway genes occurred. This likely resulted in a specific titration that drives an E2Fa-DPa^OE^-mediated pattern, counteracted by slowing DNA synthesis. This suggests that constitutive genetic or transient physiologic (stress) contexts leading to upregulation of DNA repair genes might share similar E2Fa deregulation that results in chromatin changes associated with induction of the S-phase or activation of DNA damage response proteins by AtATM.

**Figure 8:**
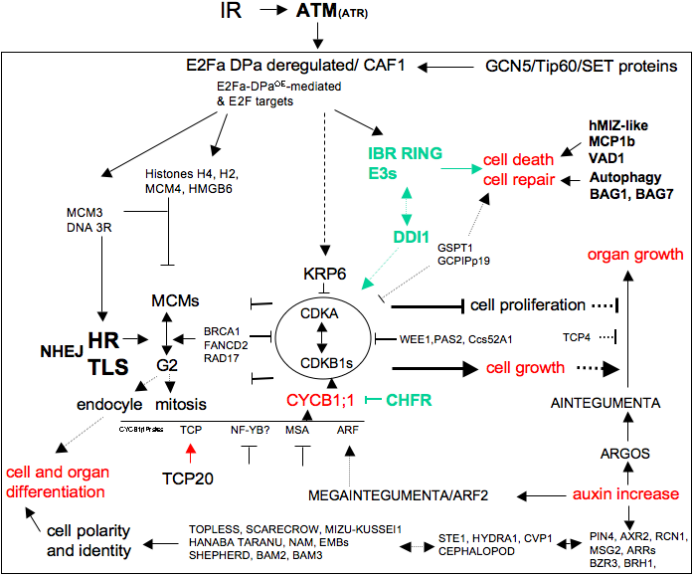
Hypothetical network of IR-regulated functions involved in development after sublethal IR. IR-ATM-mediated transcription of hallmark genes might link to the IR-inducedphenotypes of WT and *atm* (Fig. 1–3, red). Cell cycle is severely delayed after activating ATM-mediated DNA damage checkpoints by numerous inhibitors and regulators of proliferation. Cell growth and repair is enabled by upregulation of KRP6 and CYCB1;1, which regulate specific activities of CDKs. Transcriptional, translational, and/or posttranslational regulation of E2F-DPs and other TFs (cell death hMIZ-like, proliferation TCP20/TCP4), hormone (auxin)-responsive groups (ANT, ARGOS, ARF2, AXR2), development group (*e.g.*, TOPLESS, SCARECROW) in coordination with similar regulation of chromatin modifiers results in specific transcription IR- and S-phase specific patterns. Translesion synthesis, replication, and HR genes indicate an increase in replication HR repair activity. At the organ level, an increase in auxin is associated with disturbance of the whole hormones titration, cell death in the provascular system, and development patterns. Dashed arrows indicate putative links. Except for the group that includes histones, only upregulated genes are shown.

The transcription burst followed two major trends of expression in *atm*: (i)-loss or severe attenuation and delay, and (ii)-inverse and/or stochastic, as well as specific. The transcription burst followed more subtle trends in *atr* as it mainly follows the WT pattern and was poorly stochastic. These trends apparently correlate with the extent of constitutive expression in untreated *atm* and *atr*, as clusters M1 and M2 were large, whereas cluster R1 was single and included a small set of genes (Fig. 7). Due to the cellular functions represented in IR-transcript profiling, we hypothesize that IR simultaneously triggers three major types of chromatin alterations: DSB-type lesions, non-DSB-type lesions, and increased decondensation-type linked to active transcription (Figure 7). This prompted us to hypothesize that genes for which expression was IR-regulated and lost in *atm* (Fig. 7 left side) include proteins that (i) were early phosphorylated by ATM or (ii) early regulated by downstream effectors whose activity depends on early sensing by ATM, and (iii) timely and/or physically linked to ATM activity and/or to DSB sites, as they included almost exclusively cell cycle and DNA repair genes. Recently reported examples included WEE1 and CYCB1;1, which are regulated through ATM or ATR-dependent pathways [73], [165]. In a similar manner, genes that required ATM activity to establish the correct direction and timing of expression or were specifically regulated in *atm* (Fig.7 right side) were hypothesized to include targets regulated by other posttranslational regulators acting more upstream or downstream of ATM/ATR and/or further away from DSB sites, such as Tip60 and ARP4. This broad classification might help to uncover novel or known hallmark plant pathways involved in the response to IR. In an attempt to link development patterns observed in both irradiated *atm* and WT to associate molecular pathways, a schematic of the protein network that might interact at the cellular level and/or at the organ level is shown in Figure 8.

### Transcript profiling reveals putative links between cell cycle, auxin, and developmental checkpoints after DNA damage

Cell cycling arrest was largely illustrated by the decreased expression of genes that are critical for the proper maintenance of proliferative potential, developmental programs, and morphogenetic patterns. The positive regulation of KRP6 and CYCB1;1 accompanied the arrest together with numerous regulators such as the novel Arabidopsis genes hCHFR-like and ScDDI-like (Fig. 8). Ectopic expression of a dominant negative mutant of the G1/S CYCD3 (CYCD3;1Δ^OE^) showed a CYCD3.1^OE^ phenotype (override of G1/S, increased S-G2 delay, downregulation of late G2 genes) but a decreased expression of *CYCA3.2* and histone H4, and showed extensive death instead of vacuolization [77]. Such phenotypes partially overlap with the IR response, leading us to hypothesize that the transient increase in KRP6 might interact with regulated forms of CYCD3s after DNA damage. Second, IR-induced-and E2FaDPa^OE^-constitutive transcription profiles share targets involved in cell division and growth (ATPK19, CYCB1;1) and KRPs (KRP6 by IR, KRP2 and KRP3 in E2Fa^OE^) [166], [167], suggesting a mechanism by which the G1-to-S and G2-to-M transitions communicate, but are regulated by a DNA damage checkpoint. Indeed, nondegradable CYCB1;1^OE^ and KRP^OE^ plants exhibit a similar increase in cell size, featuring an IR-induced phenotype, whereas plants overexpressing *E2Fa, CYCA3;2*, or *CYCD3;1* result in more cells [168]. In human cells, CDK inhibitors include p21^CIP1^, p27^KIP1^, and INK4-type that strongly control G1/S transition, while plants have only KIP-related regulators. Human p21^CIP1^ is upregulated after IR [148] and p27^KIP1^ is an atypical E2F1 target induced by deregulated E2F1 and not only by serum activation [168]. In mice, knockout of the p27^KIP1^ gene causes hyperplasia, suggesting that p27^KIP1^ is involved in organ-size control and has a role as ‘an intrinsic timer’ in defining the extent of growth [169]. Assuming that similar functions exist in plants, this opens the possibility for KRPs and KRP6 in particular, to be regulated in a similar way after plant E2Fs deregulation. The IR-induced upregulation of the repressor of proliferation TCP4 and of TCP20, a quantitative activator of CYCB1;1 and of ribosomal gene expression might indicate a specific requirement for cell growth/repair components (sizer elements) when cells stop dividing outside M phase. Therefore, CYCB1;1 and KRP6 might respectively reveal critical “sizer and timer” components of the cell that are triggered in concert with an appropriate response to DNA damage when cells are transiently arrested. As both interact with CDKs, such a combination might help sustain the energy demand for repairing DNA and cellular components as well as for anticipating cell growth by governing the activity of CDK complexes until the cell cycle restarts. Cell size is increased and growth is reduced in both nondegradable CYCB1;1^OE^ and KRP^OE^ plants [92], similar to transient impairment of development after IR (Fig. 3). This might be related to the proliferation and growth components of the organ size checkpoint (ANT) [169] that acts as a transcription regulator [170]. Furthermore, the CYCB1;1 promoter harbors an auxin response factor (ARF)-binding site that might connect auxin titration to cell cycling and growth depending on the cell competence for division/differentiation at the time of IR. After IR, auxin increases in the columella and in the vascular system following the upregulation of many genes that are essential for the hormone response. Among them, ARF2 and ARGOS, which act upstream of ANT, might represent links between cell and organ size, cell, and organ differentiation, cell and organ fate (division, survival, senescence, death). Such a role for ARF2 would explain why the ARF2 phenotype hardly conforms to the canonical auxin response model [171]. Furthermore, SCLs, TPR, HANL, and AGL18, which are likely coregulators of cell polarity and identity might act in concert with ANT-mediated polarity of the organ, as well as with auxin-mediated pathways and cell cycle activity through the RB pathway [73], [165]. Cell death and cell repair pathways might also be related to cell cycle and DNA repair through regulators of SCF complexes and co-chaperones, or transcriptional factors such as MIZ, involved in apoptosis in human cells [125]. Cell death in provascular cells is accompanied by an auxin increase after IR. Interestingly, WEE1 kinase is expressed in such cells and in columella initials after replication block [90]. This suggests a putative link between auxin and cell lineage fate after IR, and a determinant role of kinases and phosphatases that mediate the auxin response or regulate cell cycle that were found in the IR transcription profiles. In addition, CYCD3;1Δ^OE^, WEE1^OE^, PAS2^OE^, and KRP2^OE^ plants tend to limit endoreplication, and show different levels of cell enlargement (WEE1, KRP2), suggesting that size and ploidy are not correlated, in contrast to E2Fa-DPa^OE^ plants that have increased endoreplicated cells. The transcriptional signature that transiently mimics overexpression of such genes rather argues in favor of a limited endoreplication, as E2FaDPa^OE^ targets of DNA replication were decreasing after IR. To this point, if H2B-YFP indicated premature cell differentiation and/or death (Fig. 1), other markers are required to reach definitive conclusion. Previous reports showed that IR–induced endoreplication was low in the roots compared to cotyledons and hypocotyls [63], and that roots underwent either G1/S or S/G2 arrest depending on the mutant (*lig4 vs ercc1*). The experimental set up, however, was different than ours (lethal doses on WT Landsberg ecotype imbibed seeds *vs* sublethal dose on 4-day old Col-0 seedlings in the present study), preventing a direct comparison. Together, these data indicate that the internal titration of activated cell cycle checkpoints, hormones, and developmental factors, which determine cell competence towards stemness, division, and differentiation are decisive for organ survival following IR.

### Does AtATM promote HR repair through cell cycle control?

After acute sublethal IR of division-active seedlings, our results and those reported recently [140] suggest that HR genes are essentially induced compared to NHEJ genes (several 10's - *vs* 2-fold), indicating a transient increase in HR repair, consistent with the S-G2 delay visualized by CYCB1;1. In the budding yeast, HR has a dominant role in virtually every type of DSB repair, but is not dependent on DNA replication or the presence of duplicated chromatids, but rather on Clb-CDK activity, which is required to carry out end resection, one of the earliest stages of the HR process [172], [173]. This was consistent with the absence of a clear relationship between the expression kinetics of these genes in response to IR and their regulation during the cell cycle, although many of the IR-induced genes are also regulated during the cell cycle [174]. Mammalian cell types have a different constitutive balance in HR and NHEJ efficiencies [108]. For example, mouse embryonic stem cells tend towards HR, while primary cells tend towards NHEJ. Vertebrate NHEJ-deficient *ku70* cells are extremely IR-sensitive in the G1 and early S phases, and HR-deficient *rad54* cells show a relatively flat IR sensitivity pattern, and are IR-sensitive only during the late S to G2 phases [108]. This indicated that NHEJ is the major machinery for DSB repair in the G1 phase, while HR begins to function (in addition to NHEJ) in the late S to G2 phases, and accordingly confers IR-resistance to the cells in the G2 phase (WT human cells acquire ionizing radiation-resistance as they proceed through S-phase). Moreover, in human cells defective in G1/S arrest, some of the DSBs produced in G1 and left unrepaired by XRCC4-dependent NHEJ can be processed by HR, but only in late S/G2 [175]. In Arabidopsis, *lig4* mutants arrested at G1 after IR while *ercc1* mutants arrested at G2 and were slightly less IR sensitive and more sensitive than *lig4* mutants to UV-B and to the DNA ICL-inducing agent, mitomycin C [62], [63], likely consistent with the differential use of NHEJ and HR in mammals at those phases. Whether ERCC1 is involved in HR repair of ICL-induced DSB at S-G2 in Arabidopsis like in mouse ES cells [176] is, however, still unknown. Arabidopsis *CAF-1* constitutively express HR genes, display S-phase delay, and show enhanced HR and cell death, due to increased DSBs [58], [162], [177], [178]. Furthermore, HR frequencies and RAD51 expression were naturally higher compared to KU70 in 4- d- old WT seedlings [179], indicating that HR is more predominant in division active tissues and that both mechanisms can compete towards resected ends that naturally occur during replication. Our results indicate that NHEJ genes are expressed throughout the cell cycle, while HR genes are S-phase dependent. This also suggests that IR transcription profiles mainly include oscillating genes making HR and NHEJ rather dependent on CDK/CYC activity following ATM and ATR recruitment to DSB sites, as observed in yeast and human cells [34]–[37]. This is consistent with resetting of the cell cycle after DNA damage and the transient burst in transcript changes followed by longer posttranslational regulation (CYCB1.1). In human AT-cells, 90% of DSBs are repaired by NHEJ, which appears to be ATM-independent [27], [180]. In Arabidopsis, proficiency of non-HR repair in *atm* is supported by the fact that *atm* is less sensitive to IR than is *Ku80* [140], and is also able to induce *LIG4* after IR but later than in WT. In addition, *LIG4, KU70, KU80*, and *RAD50*, are constitutively higher in *atm* (D. Camescasse & A.F. Tissier, personal communication). Therefore, the competition of NHEJ/HR towards resected ends is likely in favor of Ku-dependent NHEJ before IR in *atm*. This might be partially related to the lost regulation of PAS2 as untreated *atm* seedlings cycle slightly more quickly than WT [46]. These findings suggest that chromatin metabolism and cell cycling characteristics in *atm* are quite different from those in WT, consistent with the stochastic occurrence of misregulated clusters of genes (Fig. 7). Therefore, the hypersensitivity to DNA damage of NHEJ- proficient *atm* might originate first from cell cycle checkpoint abrogation, enabling cells to divide before complete repair than from DNA repair deficiency *per se*. Briefly, if *lig4* can be arrested by ATM activated checkpoints, *atm* cells can continue cycling using NHEJ. The ectopic division of QC and initials and lateral root initiation at a time when WT is arrested might prevent compensating for IR-DSB repair by both proficient NHEJ and residual HR (RAD51 in cluster K1 is weakly expressed) therefore enabling cell death or genetic instability as in AT-cells. Furthermore, irradiated *atm* initiated the growth of root primordial cells, although improperly positioned, indicating that cells cycle in response to the mitogenic signals independently of the development status, locating DNA damage and cell cycle checkpoints upstream. They quickly stop dividing, however, likely because of the persistence of residual breaks that might require ATM (Artemis-like) to be repaired, as even *lig4* cells, but not AT-cells, substantially recover after IR [181], [182]. In *Allium*, root tips irradiated with 40Gy X-rays underwent a caffeine-dependent G2 arrest, but IR did not prevent aberrant mitotic figures and apoptosis from occurring together with DNA contents lower than 2C, that is, a mitotic catastrophe [71]. Therefore, the stringency of plant checkpoints is likely lower compared to human, as already reported [61], [183], a feature that might be at least partially related to the absence of human INK4-type restricting inhibitors in plants. Therefore, the constitutive increase of HR genes associated with S-phase delay in mutants such as *CAF-1, tebichi*, E2Fa-DPa^OE^ or their transient increase during cell life, brings into question the role of ATM, when the level of DSBs increases due to deregulated functions and might cause genetic instability. This also underscores the importance of proficiency of DNA damage and cell cycle checkpoints in plant developmental phenotypes. To address this point, it would be helpful to compare early phenotypes of QC and initials in irradiated DNA repair- deficient mutants [62], [63].

### Putative links between development failure and transcription in irradiated *atm*

**Figure 9:**
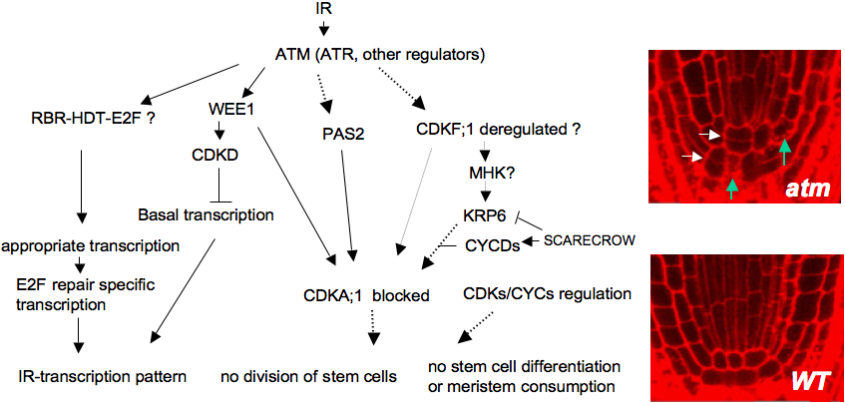
Putative hallmark regulation steps in stem cell maintenance and transcription response after IR. After IR, both the CDKF;1 pathway and WEE1 are regulated leading to inactive CDKA;1. WEE1, in addition to halting the basal transcription while disrupting in particular HDT/RBR/E2F complexes might specifically drive appropriate transcription as in human cells [152]; both are mediated by ATM. In addition, CDKF;1 is possibly deregulated, resulting in CDKA;1 inactivation. This might be promoted by stabilizing KRP6 by kinases, such as the AtMHK. Only KRP6 was upregulated after IR, however other KRPs might be involved. KRP6 might interact with CYCD3s or CYCD2-CDKs. In irradiated *atm*, transcription control might be lost due to misegulation of WEE1 and RBR/HDT and division of stem cells (white arrows) is promoted by deregulation of stem cell-maintaining RBR pathway, in addition to PAS2 deregulation and CDKF;1 misregulation. A competition between KRP/CYC/CDK and other regulators in stem cells might also lead to the differentiation of stem cells (green arrows) through ATM-dependent and independent (ATR, others) regulation of pre-existing factors at the time of IR [140]. After ectopic division due to checkpoint abrogation, atm stem cells accumulated high amounts of unrepaired DNA and/or small amounts of DNA lesions that require ATM-Artemis-related DNA repair functions resulting in meristem consumption similar to irradiated human ataxia telangiectasia-cells. Dashed arrows indicate unknown regulators or pathways. Other regulators shown in Fig. 7 were not shown and images are extracted from Fig. 2.

The “protection” of initials in irradiated WT followed by the restart of meristem division several days post-IR was opposite the mixed pattern of dividing/differentiating stem cells and QC that occurred 1 d post-IR and preceded meristem consumption in irradiated *atm*. At the same time, the WT transcriptional burst was absent or inverse in *atm*. WEE1 kinase regulates basal transcription through CDKD inhibition [184] and controls CDKA;1 activity by ATM (ATR)-mediated regulation [90]. Thus, the absence of WEE1 regulation might explain part of the radiation-resistant transcription of *atm*. Another possibility is that the radiation-resistant transcription of *atm* is caused by ATM-mediated deficient disruption of the histone modifiers/TFs/regulators chromatin complexes required for appropriate transcription after IR (Fig. 9). The failure of *atm* to regulate hormone-responsive genes and especially PIN4 might increase auxin levels in *atm* root tips due to the absence of a focused PIN4-driven auxin sink in the first columella tier [185], and therefore influence stem cell fate. Determining whether such auxin-responsive genes and WEE1 kinase, which is specifically induced in the vascular system and is located close to the QC after replication block [90], are co-regulated after IR would help to identify morphogenetic pathways. Clearly, ATR, mitogen activated protein kinases, and CDKs and numerous IR-upregulated kinases not yet characterized might also regulate such a network, as in human cells [22], [37]. Indeed, AtATR drives the stabilization of CYCB1;1 in the *atm* and WT meristematic zone after IR [140], indicating that proteins existing at the time of IR are immediately regulated, and likely explains the similarity of prematurely differentiated cells in WT and *atm*. What happens in stem cells, however, is still unknown. The mRNA levels of several G1, but not G2, regulators change in *scr* mutants, indicating a peculiar role of G1 regulators in the RBR/SCR-mediated pathway that controls stem cell maintenance [73]. CDKF1;1, which strongly regulates the cell cycle [184], might also be misregulated in *atm* stem cells after IR in a manner similar to that observed when CDKF;1 ectopic expression leads to a reduction in CDKA;1 activity and differentiation of columella stem cells [74]. CDKF;1 is a functional homologue of yeast CAK1 that regulates CDC28/CDK activity during meiotic differentiation [186] through the kinase IME2/PIT1, which stabilizes Sic1p, the functional orthologue of human p27^KIP1^ [187]. In humans, the G0 checkpoint nuclear kinase DYRK1 has a critical role in growth arrest, transcription, and cell survival, including stabilization of p27^KIP1^, destabilization of CYCD1, and relocation of HDAC/TF and p21^CIP1^ [188]. Among the IR-regulated kinases, the male germ cell-associated kinase (MAK), AtMHK, was the only kinase containing the characteristic TEY motif that essentially aligns with the T-loop of SpPIT1 (data not shown) among 10 MAK/ICK sequences from humans and yeasts [189], and OsYakA-like was the only DYRK-type kinase [188]. AtMHK is an orthologue of the Cdc2-related BvCRK2 that is implicated in cell division and the early events of differentiation, when *CDC2* is expressed in the majority of cells in the developing organ. Because DYRK-and MAK-type kinases are involved in crucial steps of tissue development, it is possible that stabilization/regulation of KRP6/KRPs by AtMHK and/or YakA occurs after DNA damage in arrested cells. This would help prevent a mixed division and differentiation status of the stem cells before complete repair, as WT was arrested while *atm* was dividing (Fig. 9). Also, this could partially explain why columella stem cells differentiated after CDKF;1 ectopic expression [74]. Finally, CDKA;1 and RBR pathways affect male gametogenesis and female gametophytes, respectively, in Arabidopsis [88]. Because *atm*, but not *atr*, is partially impaired in meiosis and *atm atr* is fully sterile [46], [47], investigating the roles of AtATM, AtATR, AtMHK, YakA, and similar pathways in meiotic cells and somatic cells after DNA damage would help to elucidate the crosstalk between cell cycle and DNA damage checkpoints activated by genotoxins and IR or by natural DNA lesions. As KRP and SCR activities act upstream of the RBR pathway, future studies will help to decipher the roles of AtATM in concert with the TOPLESS, SCARECROW, PLETHORA, and SHORT ROOT pathways in cell stem fate when there is increased DNA damage [73], [133].

During revision of the manuscript, De Schütter *et al.* [90] reported ATM/ATR-mediated regulation of WEE1 kinase, and Culligan *et al.* [140] reported a similar transcriptional response and demonstrated that CYCB1;1-GUS increased while CYCB1;2-GUS decreased after IR, consistent with the transcriptional pattern we observed here. CYCB1;1 stability was ATR-mediated, showing that posttranslational and/or translational regulation of pre-existing proteins/mRNAs occurred in both *atm* and WT. These data are highly complementary.

## Material and Methods

### Tissue preparation and confocal laser scanning microscopy

For propidium iodide (PI) staining of live root cell walls, seedlings were submerged in 5 mg/L PI for 1 min and rinsed; the roots were then mounted on slides in water. For pseudo-Schiff staining of roots, seedlings were fixed in a 50% methanol, 10% acetic acid solution and kept overnight at 4°C, washed twice in water, and treated with 1% periodic acid for 30 min at room temperature. Seedlings were washed again and incubated for 2 h in fresh Schiff's reagent solution (1.9 g sodium metabisulphite, 97 mL water, and 3 mL 5N HCl) supplemented with 20 µg/mL PI. Seedlings were then washed again and cleared for 1 h in a chloral hydrate solution (80 g in 30 mL water). Finally, roots were cut and mounted on slides in Hoyer's solution (30 g gum arabic, 200 g chloral hydrate, 20 g glycerol in 50 mL water) clarified by centrifugation. For viability staining of root tip cells, seedlings were incubated 30 min in a 5 µg/mL fresh fluorescein diacetate (FDA) solution, washed with water and incubated 3 min in a 20 µg/mL PI solution. Confocal laser scanning microscopy was performed using an Olympus Fluoview microscope equipped with an argon-HeNe laser. Excitation and emission wavelengths were 488 nm and 505–530 nm, respectively, for FDA, GFP, and YFP, and those for PI were 543 nm and 585 nm, respectively. Binocular imaging of CYCB1.1-GFP in roots was performed directly in the culture Petri dish using a stereomicroscope (Leica MZFLIII, Germany) and a Spot Advanced version 4.0.1 (Diagnostics Instruments Inc, Sterling Heights, MI) for image acquisition. Image processing was completed using Photoshop 7.0 (Adobe, San Jose, CA).

### Seedling culture and irradiation, mutant lines, and total RNA extraction

Seedlings were cultured in vivo in Petri dishes as described previously[54]. Wild-type, *atm2*−/− (*atm*, Salk-6953), and *atr2*−/− mutants (*atr*, Salk 32841) were in ecotype Col-0 [46], [47]. Histone H2B::YFP, CYCB1;1::GFP, and DR5::GFP lines were kindly provided by Dr. F. Berger and Dr. P. Doerner [64], [66]. Surface sterilized seeds (80) were sown in two rows per Petri dish, vernalized 2 d at 4°C, vertically grown with a 14-h photoperiod at 22°C (10 h dark at 18°C). Two hours after turning on the light in the culture chamber, 4-d-old seedlings (developmental stage 0.7–1 according to [190]) were given a single dose of 100 Gray γ-rays with a ^60^Cobalt source (22 Gray min^−1^). Control plants were placed in the dark close to the IR platform. After IR, irradiated and control seedlings were returned to the culture chamber for up to 5 h, then harvested 0.75 h-, 1.5 h-, 3 h-, and 5 h after IR, and immediately frozen in liquid nitrogen and stored at −80°C. Root samples were harvested 1 h after IR by discarding the apical part of the seedlings.

### Transcriptome studies

The microarray analysis was performed at the Unité de Recherche en Génomique Végétale (URGV), (UMR INRA 1165 - CNRS 8114) using the Complete Arabidopsis Transcriptome MicroArray (CATMA) [191], [192] containing 24 576 gene-specific tags (GSTs) from Arabidopsis [193]. The spotting of the GST amplicons on array slides and the array analysis process were previously described [194]. Total RNA was extracted from 4 –d -old seedlings or roots with Trizol (Invitrogen, Carlsbad, CA) according to the manufacturer's protocol and samples were hybridized as shown in Figure 5. Each experiment was performed in duplicate. For each comparison, one technical replication with fluorochrome reversal was performed for each pool of RNA. RNA integrity was checked with the Bioanalyzer from Agilent (Waldbroon, Germany). cRNAs were produced from 2 µg of total RNA from each sample with the Message Amp aRNA kit (Ambion, Austin, TX). Then, 5 µg of cRNA was reverse transcribed with 300 U of SuperScript II (Invitrogen) and cy3-dUTP or cy5-dUTP (NEN, Boston, MA) for each slide. Samples were combined, purified, and concentrated with YM30 Microcon columns (Millipore, Billerica, MA). Slides were prehybridized for 1 h and hybridized overnight at 42°C in 25% formamide. Slides were washed in 2X SSC+0.1% SDS for 4 min, 1X SSC for 4 min, 0.2X SSC for 4 min, and 0.05X SSC for 1 min, and dried by centrifugation. Two hybridizations (one dye-swap) were performed. The arrays were scanned on a GenePix 4000A scanner (Axon Instruments, Foster City, CA), and images analyzed by GenePix Pro 3.0 (Axon Instruments).

### Statistical analysis of microarray data

Experiments were designed in collaboration with the Statistics team of the URGV. The statistical analysis was based on one dye-swap (*i.e.*, two arrays each containing 24 576 GSTs and 384 controls). The controls were used for assessing the quality of the hybridizations, but were not included in the statistical tests or the graphical representation of the results. For each array, the raw data comprised the logarithm of median feature pixel intensity at wavelengths of 635 nm (red) and 532 nm (green). No background was subtracted. In the following description, log ratio refers to the differential expression between two conditions; log2 (red/green) or log2 (green/red), according to the experimental design. An array-by-array normalization was performed to remove systematic biases. First, we excluded spots that were considered badly formed features. Then, we performed a global intensity-dependent normalization using the LOESS procedure to correct for dye bias. Finally, for each block, the log-ratio median calculated over the values for the entire block was subtracted from each individual log-ratio value to correct print tip effects on each metablock. To determine differentially expressed genes, we performed a paired t-test on the log ratios, based on the assumption that the variance of the log-ratios was the same for all genes. Spots displaying extremes of variance (too small or too large) were excluded. The raw P values were adjusted by the Bonferroni method, which controls for the Family Wise Error Rate. We used the Bonferroni method (with a type I error equal to 5%, Bonferroni p-value≤0.05) to control for false positives due to multiple comparisons [195]. The data were deposited in Array express according to the MIAME standards (Accession number E-MEXP-780 (http://www.ebi.ac.uk/arrayex-press/experiments/E-MEXP-780)).

### Real-time quantitative PCR

Primers of genes of interest (Table S1-Primers) were designed with Primer 3 (http://fokker.wi.mit.e-du/cgi-bin/primer3) and checked with Amplify (http://engels.genetics.wisc.edu/amplify/) before synthesis. Expression changes were calculated as described in Lafarge & Montané [54].

### Gene clustering

Excel tables containing genes with at least one statistically relevant ratio (Bonferroni p-value≤0.05) among seedling or root experiments were provided by the URGV platform. Manual clustering was performed using a color code that only tags ratios fulfilling the Bonferroni criteria. K-means clustering was also performed using all ratio-values regardless of their Bonferroni p-value with Genesis software set with default parameters and run for 8 clusters. Images were extracted from Genesis or Excel files with Capture version 1.3 and Aperçu version 3.0.8 (Apple Computer, Inc., Cupertino, CA). Methodology of gene clustering for seedling and root experiments is detailed in Fig. S3.

### Transcriptome data compilation and Venn diagram design

We created a complete list (Table S3-A) of genes from Tables S1 and S2. From this list, a tool developed using the php language that allowed us to add data from the literature, extract sets of genes based on specific criteria, generate intersections between sets, and create input files for drawing Venn Diagrams that were designed with the R software (The R Foundation for Statistical Computing Version 2.2.1), and the LIMMA library (Linear Models for Microarray Data) from the Bioconductor project (www.bio-conductor.org/).

### Gene classification into functional categories

Gene information other than that found in the TAIR database (http://www.arabidopsis.org/) was imported from the SGD (www.yeastgenome.org/) and NCBI (www.ncbi.nlm.nih.gov/) databases. Searches for conserved protein domains (www.ebi.ac.u-k/InterProScan/) and/or homology to genes from other eukaryotes, as well as in the literature, were performed to help identify novel genes and to help classify genes into functional categories. Classification of genes into 9 groups based on basic macromolecular metabolism and cellular processes and/or compartments was performed according to the rationale developed for C*aenorhabditis elegans* and classification of Arabidopsis genes required for embryo development [196]. The functional classes were: (1) DNA, chromosome and chromatin metabolism; (2) RNA metabolism including splicing, processing, and RNA binding; (3) protein synthesis, modification, and proteolysis, including translation, folding, and ubiquitin proteasome system; (4) metabolism including energy production, primary, and secondary metabolism; (5) cell biology and cellular structure, including cell cycle genes, cell receptors, cytoskeleton, cell wall metabolism, molecule transport, protein trafficking, membrane protein, vesicle regulation, and cell polarity; (6) gene specific transcription, including transcription factors; (7) stress-induced genes; (8) signalling pathways, including kinases and phosphatases and uncharacterized hormone-responsive genes. The unknown function class (9) included predicted proteins (i) whose mRNAs hybridized with a single CATMA probe (clusters K1M-K8M, M1M-M8M, R1M-R6M), (ii) and those with domains of unknown function, and (iii) with no significant matches in any database.

## Supporting Information

Figure S1 Fluorescence micrographs of WT live root tips after IR.
(0.52 MB TIF)

Figure S2 Time-course of WT primary root growth after IR.
(0.12 MB TIF)

Figure S3 Gene clustering.
(0.42 MB PDF)

Figure S4 Data validation by rt-qPCR
(0.24 MB PDF)

Figure S5 Overlap of K3 and M5 genes and functional classes distribution.
(0.25 MB PDF)

Figure S6 General characteristics of radiomodulated genes in roots and seedlings.
(0.22 MB PDF)

Table S1 Time-course of gene expression in wild type and atm seedlings after IR.
(0.66 MB XLS)

Table S2 Radiomodulation of transcripts in WT, atm, and atr roots.
(0.80 MB XLS)

Table S3 Complete list and expression characteristics of radiomodulated genes in Arabidopsis WT, atm, and atr roots and seedlings.
(0.64 MB XLS)
