# Supplementary figures and images for: ATM-Mediated Transcriptional and Developmental Responses to γ-rays in Arabidopsis
Source: PLoS One. 2007 May 9;2(5):e430. doi: 10.1371/journal.pone.0000430 (PMC1855986; doi:10.1371/journal.pone.0000430)

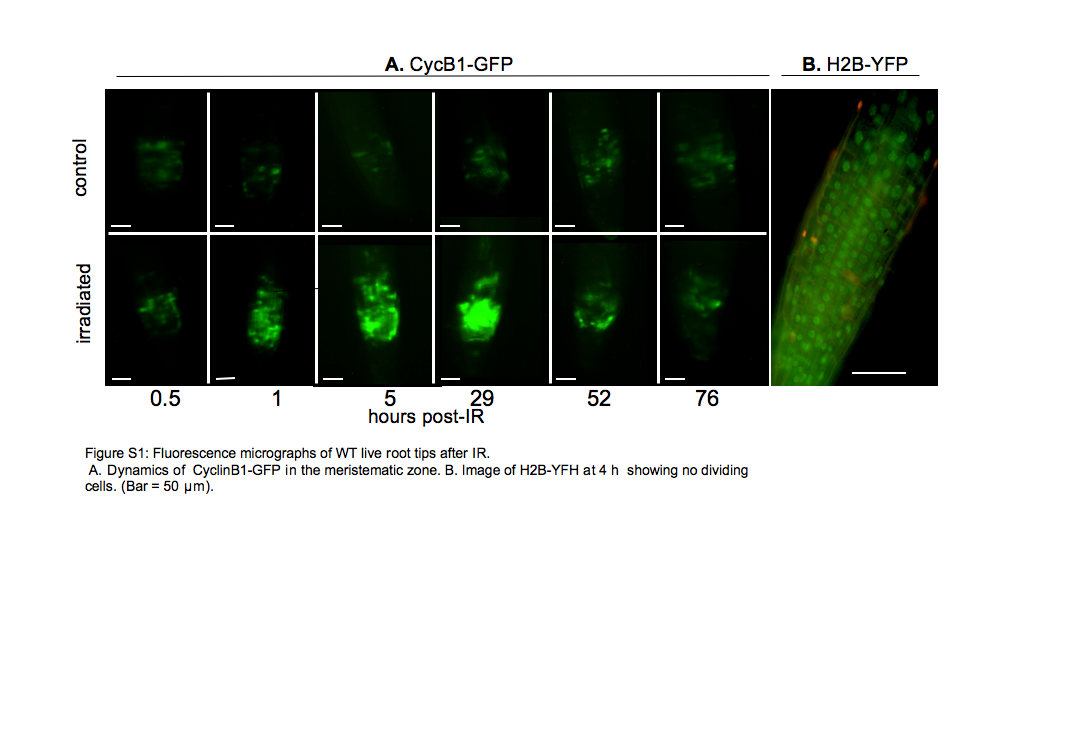

Supplement: Figure S1 — Fluorescence micrographs of WT live root tips after IR. (0.52 MB TIF) [file pone.0000430.s001.tif]

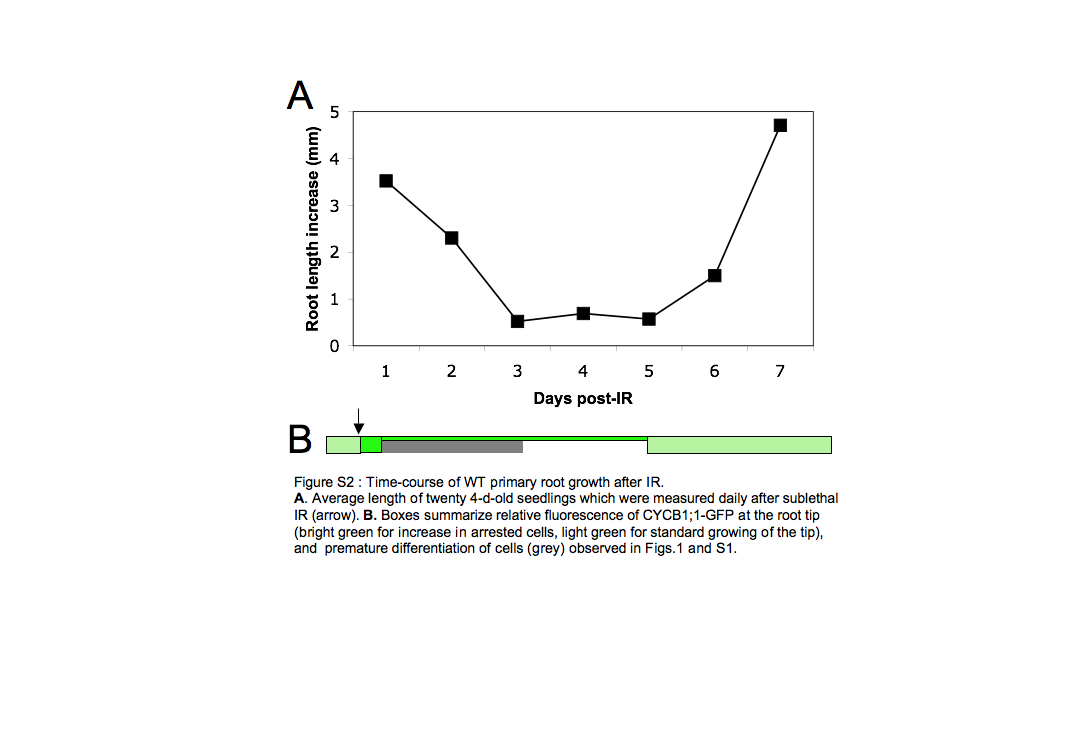

Supplement: Figure S2 — Time-course of WT primary root growth after IR. (0.12 MB TIF) [file pone.0000430.s002.tif]
