# Supplementary material for: ATM-Mediated Transcriptional and Developmental Responses to γ-rays in Arabidopsis
Source: PLoS One. 2007 May 9;2(5):e430. doi: 10.1371/journal.pone.0000430 (PMC1855986; doi:10.1371/journal.pone.0000430)
Supplement: Figure S3 — Gene clustering. (0.42 MB PDF) [file pone.0000430.s003.pdf]

Figure S3. Gene clustering.

A- *Clustering of seedling genes*. Expression profiling of Arabidopsis seedlings was performed as shown in Figure 4-A. Response to IR of approximately 300 seedlings was compared to that of the non–irradiated controls at each time point. Genes showing at least one differential expression in the WT and *atm* seedlings were obtained by extracting from the complete list of genes those with at least one ratio with a Bonferroni p-value ≤0.05 (Tables S1). Therefore, this treatment allowed for the detection 1.5-fold changes in gene expression because the average lower threshold value among all experiments was 0.65+/- 0.15, corresponding to a minimal theoretical modulation of 2 exp (+/- 0.65). **Manual clustering:** From that table, genes were organized in two steps. They were first sorted according to the direction of their regulation (up or down) in WT and then according to the sense of radiomodulation in *atm*. Clusters K1-K3 displayed genes that were upregulated in WT, and were either upregulated (K1), invariant (K2), or downregulated (K3) in *atm*, regardless of their time of expression post-IR. Clusters K4-K6 displayed downregulated genes in WT and also either downregulated (K4), or invariant (K5), or upregulated (K6) in *atm*. Clusters K7 and K8 included genes that were not significantly changed in WT, and whose expression was up –or downregulated in *atm*, respectively. Table S1.1 displays genes of all clusters excepted those identified by a CATMA probe that hit multiple (M) AGI loci (usually two contiguous loci), which are listed in Table S1.2. **K-means clustering:** The Genesis software (Sturn A, Quackenbush J Trajanoski Z (2002) Genesis: cluster analysis of microarray data. Bioinformatics 18: 207-8.) was obtained from the Graz University of Technology (http://genome.tugraz.at).

***B- Clustering of root genes*.** According to the experimental design (Fig. 5-A and C), four combinations of hybridization of the root samples were compared, thereby providing auto-validated data through the following rationale: (i) a gene experiencing a statistically significant change (ratio with Bonferroni p-value ≤0.05 in colored cells of Tables S2) in one of the four combinations should also experience at least one other significant change among the other three combinations, and (ii) when two significant changes occurred, both ratios should have similar values. Therefore, genes modulated with Bonferroni p-value≤0.05 in at least two of the four combinations were highly relevant (clusters M1-M4). Ratio-values were not similar for both combinations in all cases, however, resulting in poor linear scaling of experimental ratios *vs* each other (Fig. S6-A and C). Linear scaling was obtained when all ratio values were taken (Fig. S6-B, D), indicating a lower significant threshold value of 0.4. This was used to cluster genes with significant expression changes in only one of the four combinations and fulfilling that criterion in another condition, in order to maximize the classification of genes and to avoid a too stringent selection before the comparison of root data sets (clusters M and R) with seedling data sets (clusters K) shown in Fig. 6. As in the time-course experiments, genes were first clustered according to the direction of radiomodulation in WT [invariant (grey cells), up- (red cells), and down- (green cells) regulated in (B), and then in the mutant (C), etc.].

C- Determination of experimental threshold ratio-value.

Clusters M1 and M3 included 256 and 148 genes, respectively, that were significantly changed (Bonferroni p-value ≤0.05) in only two combinations (A and D for M1 genes) and (B and D for M3 genes), respectively (Table S2.2). The threshold-values of ratios (Bonferroni p-value≤0.05) in A, B, C, D were 0.70, 0.77, 0.69, and 0.78, showing the range of lowest statistically significant ratio value between independent experiments with four samples hybridized twice each (Fig 5-B). Plots **(A)** and **(C)** show the poor linearity between experimental ratio values in D *vs* experimental ratio values of the other condition showing a statistically significant change *i.e.,* A for genes of cluster M1, or B for genes of cluster M3. Plots **(B)** and **(D)** show better linearity of experimental ratio values (Re) *vs* calculated ratios (Rc) in D obtained by considering the ratio values of the other three conditions independently of the p-value. For M1 genes, Rc = Re (A) – Re (B) + Re (C). For M3 genes, Rc = Re (B) – Re (C) – Re (A). For each cluster, linear scaling was strongly increased (R^2 higher) when

ratios in all conditions were included, showing that the stringency of statistical treatment was high and tended to generate false negative data for low ratios. Values of Y-intercepts in plots A and C were 0.63 and 0.4 for clusters M1 and M3, respectively. Correlation coefficients of linear regression curves in each plot showed that Re (condition 4) better fit the calculated ratios. Therefore, the Y-intercept of linear regression curves in plots (A) and (B) provided minimal threshold values of 0.63 and 0.4 that ensured linearity between ratio values in the four conditions within the experimental design. To complete gene classification, the 0.4 threshold value was used to cluster genes that significantly change in only one combination or two significant changes associated with too distant ratios values (Tables S2).
Conditions 1, 2 and 4 are, respectively, A, B, and D in Tables S2;1 and S2.2.

A

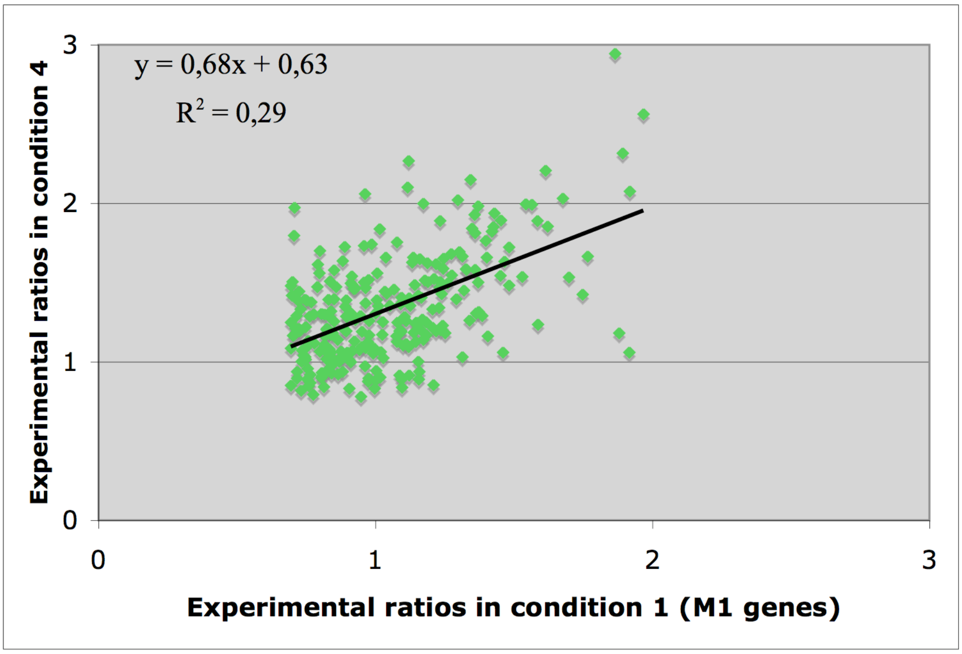

B

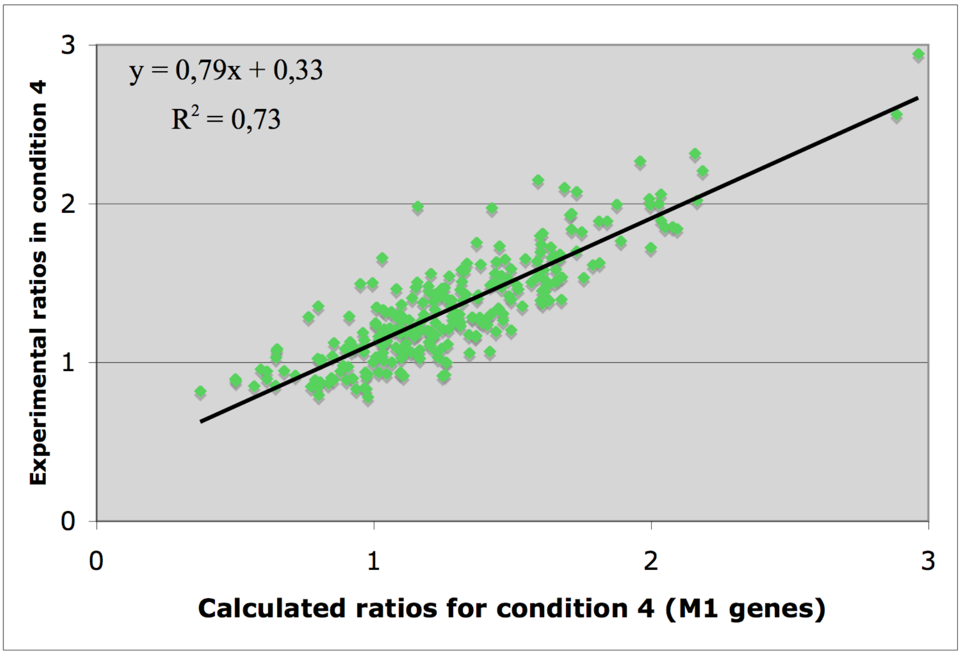

C

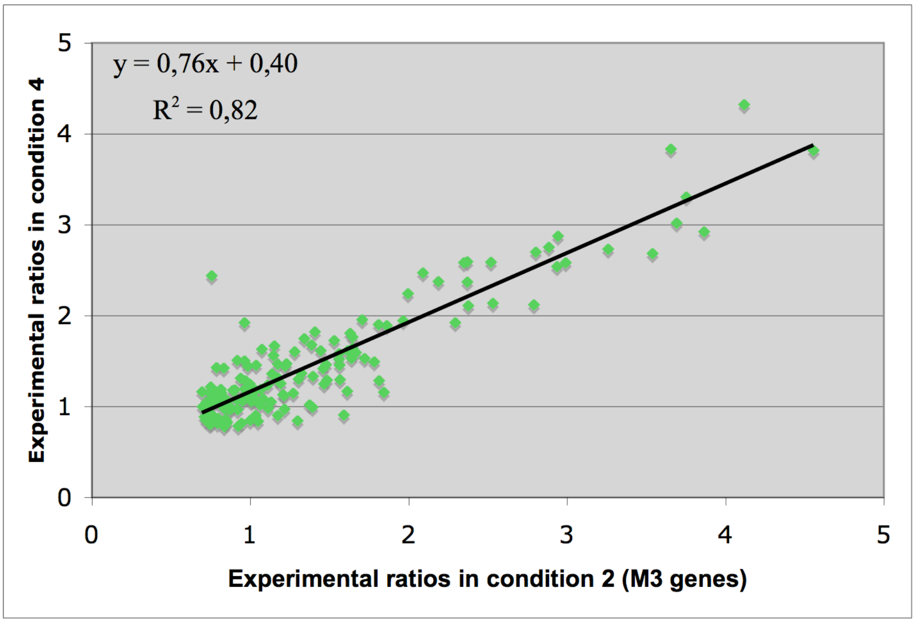
y = 0,76x + 0,40
R^2 = 0,82
Experimental ratios in condition 4
Experimental ratios in condition 2 (M3 genes)
0
1
2
3
4
5

D

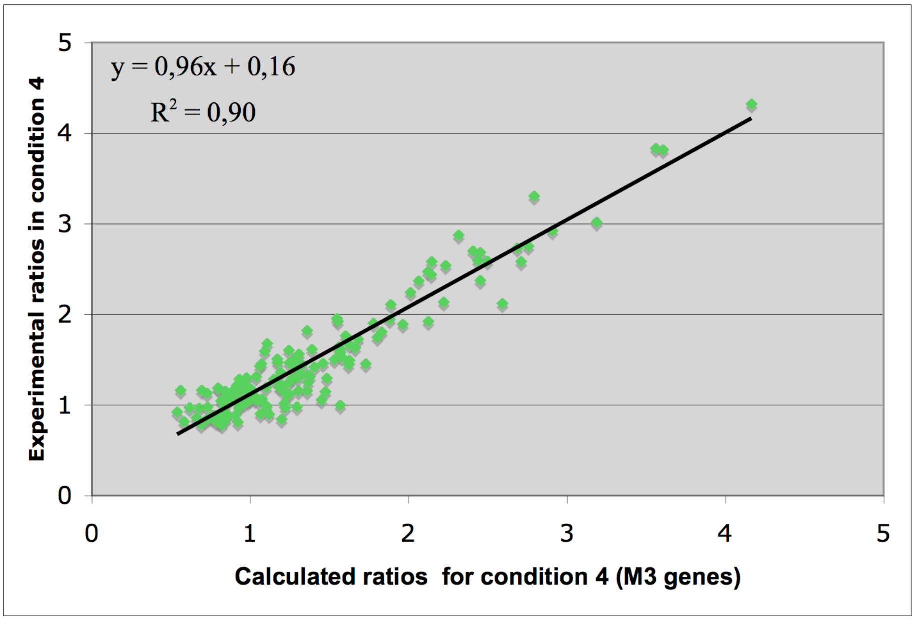
y = 0,96x + 0,16
R^2 = 0,90
Experimental ratios in condition 4
Calculated ratios for condition 4 (M3 genes)
0
1
2
3
4
5
