# Supplementary material for: ATM-Mediated Transcriptional and Developmental Responses to γ-rays in Arabidopsis
Source: PLoS One. 2007 May 9;2(5):e430. doi: 10.1371/journal.pone.0000430 (PMC1855986; doi:10.1371/journal.pone.0000430)

Figure S4. Data validation by rt-qPCR.
(A) Fold-induction by rt-PCR *vs* fold induction by microarrays [2 exp(ratio)]. The expression levels of 51 genes listed in Table S6 were examined. (B) Time-course of the expression of three upregulated genes in *atm* and WT.

A

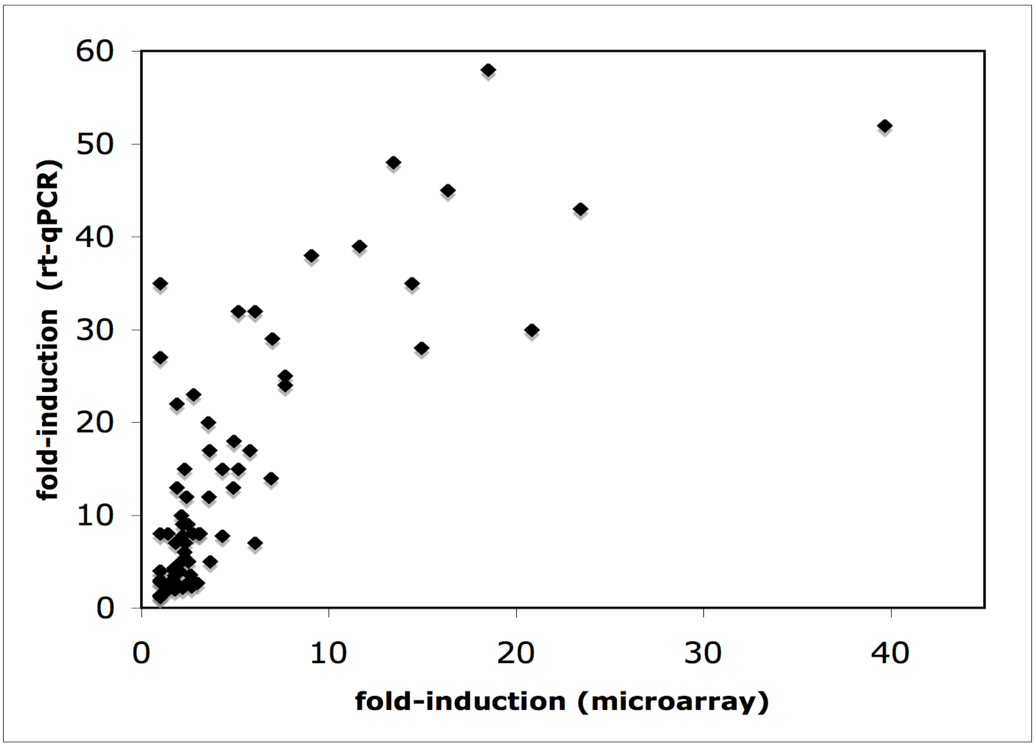

B

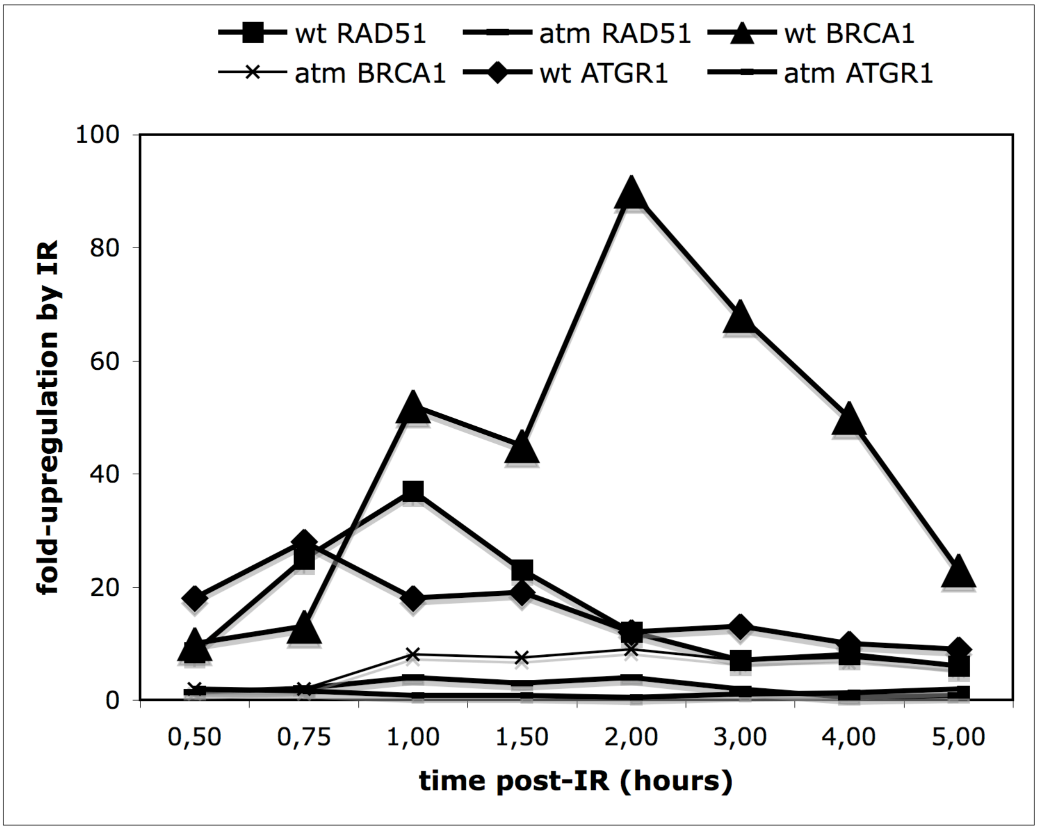

Supplement: Figure S4 — Data validation by rt-qPCR (0.24 MB PDF) [file pone.0000430.s004.pdf]
