# Supplementary material for: ATM-Mediated Transcriptional and Developmental Responses to γ-rays in Arabidopsis
Source: PLoS One. 2007 May 9;2(5):e430. doi: 10.1371/journal.pone.0000430 (PMC1855986; doi:10.1371/journal.pone.0000430)
Supplement: Figure S5 — Overlap of K3 and M5 genes and functional classes distribution. (0.25 MB PDF) [file pone.0000430.s005.pdf]

Figure S5. Overlap of K3 and M5 genes and functional classes distribution.

A- clusters K3 and M5 largely overlap. B- Cluster M5 is enriched in metabolism-related genes (class 4) while cluster M3M7 is enriched in DNA and chromatin metabolism-related genes (class 1) and transcription factors (class 6).

A

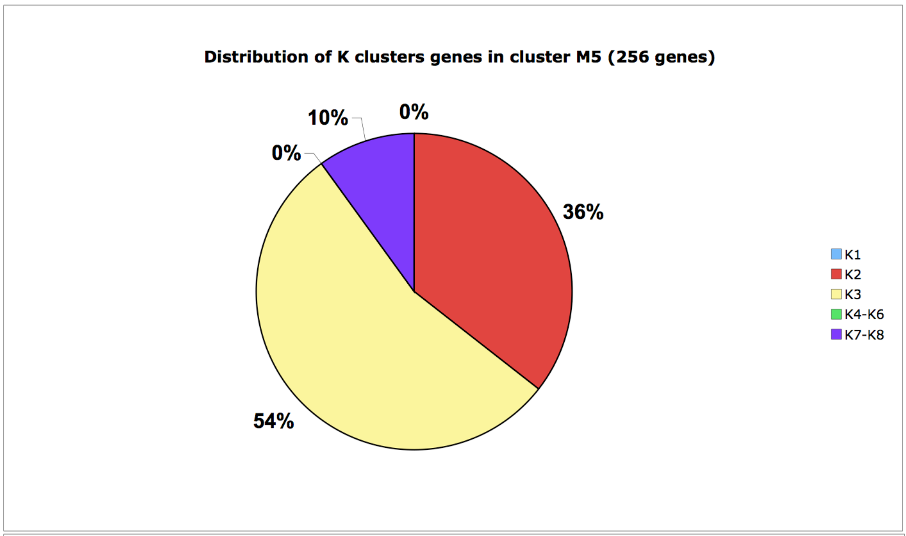

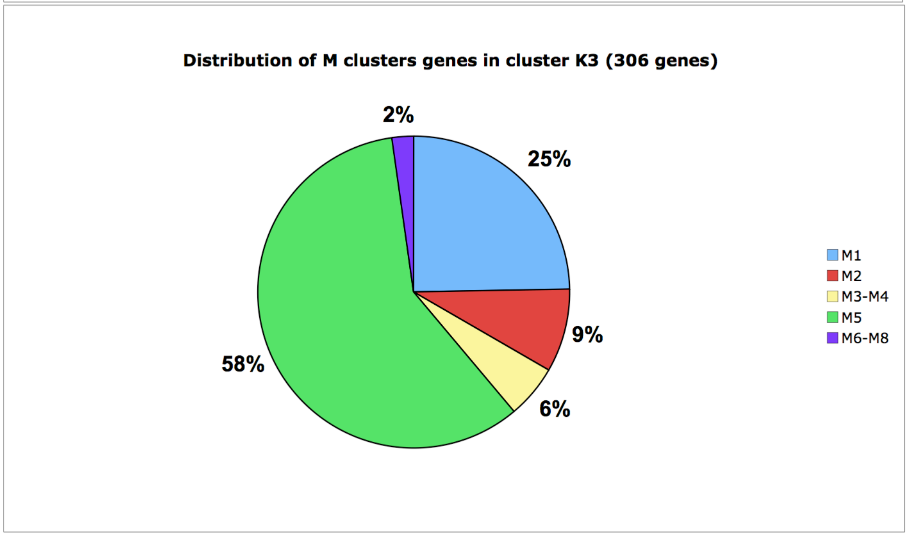

B

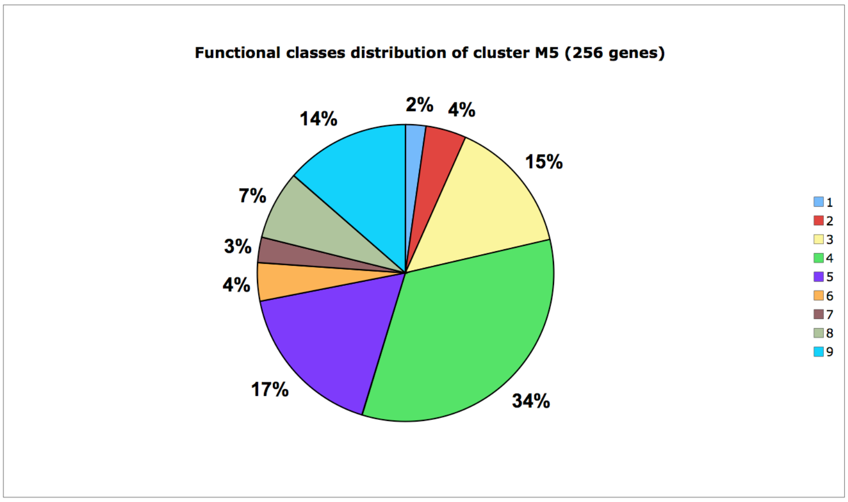

Functional classes distribution of cluster M5 (256 genes)
2%
4%
15%
34%
17%
4%
3%
7%
14%
1
2
3
4
5
6
7
8
9

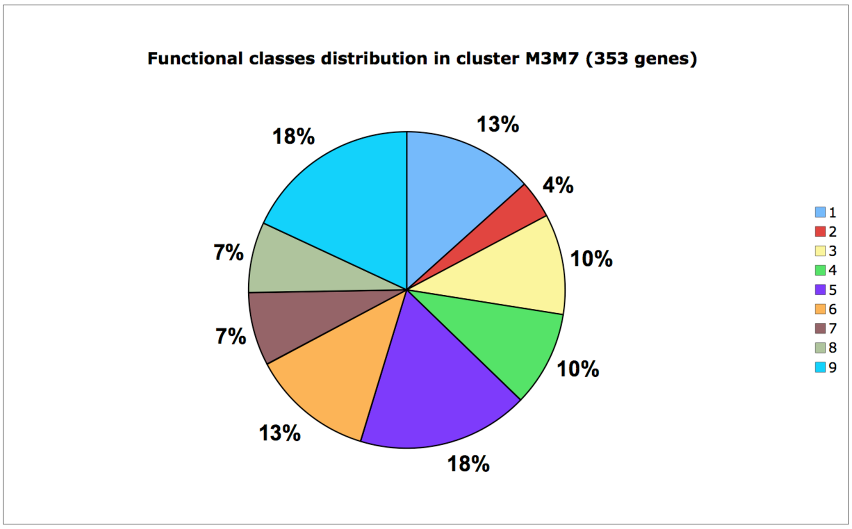

Functional classes distribution in cluster M3M7 (353 genes)
13%
4%
10%
10%
18%
13%
7%
7%
18%
1
2
3
4
5
6
7
8
9
