# Supplementary material for: ATM-Mediated Transcriptional and Developmental Responses to γ-rays in Arabidopsis
Source: PLoS One. 2007 May 9;2(5):e430. doi: 10.1371/journal.pone.0000430 (PMC1855986; doi:10.1371/journal.pone.0000430)
Supplement: Figure S6 — General characteristics of radiomodulated genes in roots and seedlings. (0.22 MB PDF) [file pone.0000430.s006.pdf]

Figure S6. General characteristics of radiomodulated genes in roots and seedlings.
Because the transcriptional IR-response was concomitant with cell cycle delay, we assume it might include decisive gene information enabling a comprehensive view of the developmental phenotypes observed in WT and *atm* (Fig. 1-2). Therefore, the transcriptional content was upgraded by (i) an additional description of individual genes to identify putative orthologues of mammals and yeasts and or gene descriptions that were not in the *Arabidopsis* databases, (ii) a broad classification of the large number of radiomodulated genes into 9 categories dealing with major components of cell structure and biology, and (iii) the import of expression characteristics such as cell cycle peaking, root subzone(s)-expression (LEDs), or sets of transcripts constitutively or transiently expressed in mutants that are hypersensitive to DNA damaging agents (*CAF-1, Ku80, tebichi*, E2Fa-DPa^{OE}). All data are in Table S3.

(A) Overview of analyzed unique genes**.** « Seedling » genes were only in clusters K1-K3 and K7 and clusters K4-K6 and K8 « Root » genes were only in clusters M3 and M7 and R2-R4 and clusters M4 and R5. « Root and seedling » included subsets of genes either up or downregulated the same way in seedlings and roots (intersection of K1-K3 and K7 with M3 and M7, and R2-R4 or intersection of K4-K6 and K8 with M4, and R5). Genes inversely regulated in roots and seedlings and genes K1M-K8M, LxM, RxM were discarded. **(B).** LED distribution. « Reference » was the relative distribution of the 5717 genes in each LED characterized by Birnbaum et al. 2003. **(C)** Cell cycle phase-peaking distribution. « Reference » was the relative distribution of the 1462 genes peaking in each phase of the cell cycle characterized by Menges et al. 2005. **(D)** and **(E)** Functional distribution of upregulated and downregulated genes, respectively. 1 Chromatin, DNA, chromosome; 2 : RNA; 3 : protein fate ; 4 : general metabolism ; 5 : cell structure and physiology; 6 : transcription ; 7 stress and hormone-responsive; 8 : signaling ; 9 : unknown function (see Material and methods). Down radiomodulated genes in both “root” and “seedlings and roots” genes were enriched in LED 8-type and M phase-type, while they declined in LEDs6-7-type or G1 and S phase-type genes. Concomitantly, upregulated genes were enriched in S-phase genes without bias to a LED-type. LED8 and LEDs6-7 spanned all radial zones but peaked in the broad meristematic zone, and all root zones located above the meristematic zone. As LED8 and LEDs6-7 included genes involved in nuclear organization, cell cycle, and mitosis, or encoding kinases and transcription factors (TFs) associated with cell maturation, their IR-induced patterns indicate that all root zones experienced a general decline of proliferation and differentiation active in untreated plants, consistently with the penetrability and the toxicity of gamma rays. Concomitantly, the S-phase-type enrichment in upregulated genes show that IR also induced growth-promoting genes, therefore indicating either the asynchrony of cells, or both cell cycle arrest and preparation for re-entry into the cell cycle in the same cell. Upregulated “seedling” genes did not reveal the bias observed in roots, and were strongly enriched in metabolism genes (Class 4 in D-E), consistent with the specific physiology and infrequent cell division in cotyledons after germination compared to division-active roots.

A

	IR-regulation	Analyzed	LED 1-8	Cell cycle phase peaking	LED 8 and M phase peaking
seedling	up	888	303	64	2
	down	456	187	57	11
root and seedling	up	166	58	13	0
	down	61	38	26	13
root	up	255	85	23	2
	down	113	45	29	10
Total no of genes		1940	717	213	38

B

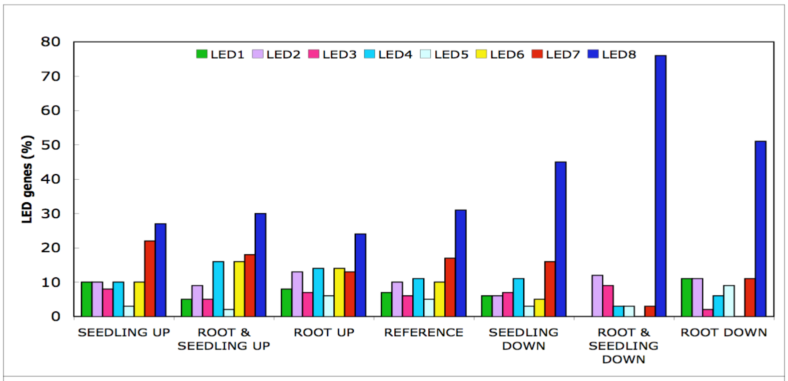
LED1 LED2 LED3 LED4 LED5 LED6 LED7 LED8
LED genes (%)
0 10 20 30 40 50 60 70 80
SEEDLING UP
ROOT & SEEDLING UP
ROOT UP
REFERENCE
SEEDLING DOWN
ROOT & SEEDLING DOWN
ROOT DOWN

C

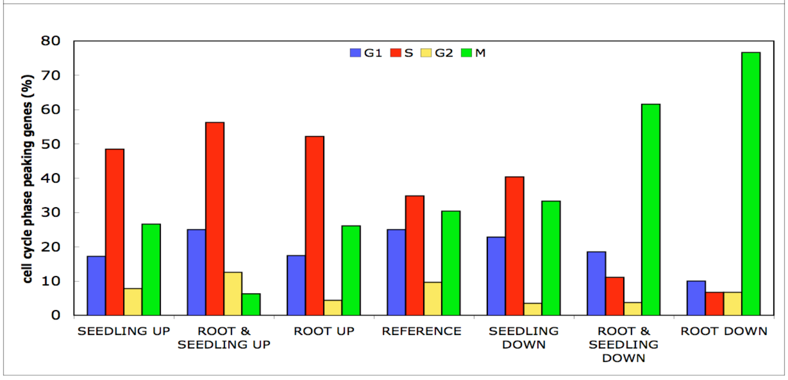
G1 S G2 M
cell cycle phase peaking genes (%)
0 10 20 30 40 50 60 70 80
SEEDLING UP
ROOT & SEEDLING UP
ROOT UP
REFERENCE
SEEDLING DOWN
ROOT & SEEDLING DOWN
ROOT DOWN

D

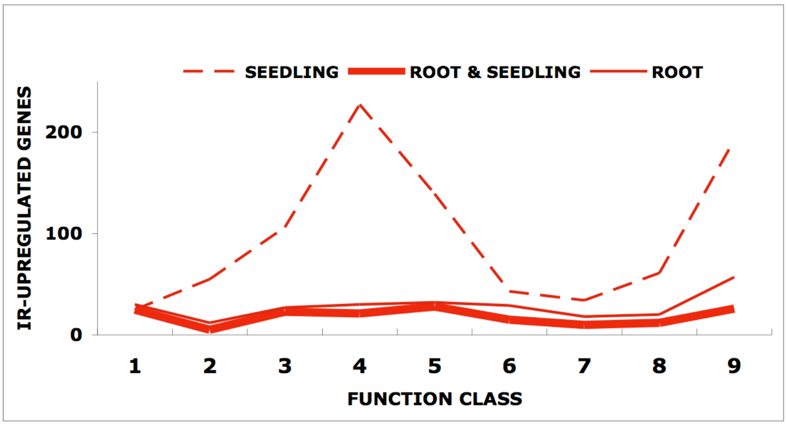
SEEDLING
ROOT & SEEDLING
ROOT
IR-UPREGULATED GENES
0
100
200
1 2 3 4 5 6 7 8 9
FUNCTION CLASS

E

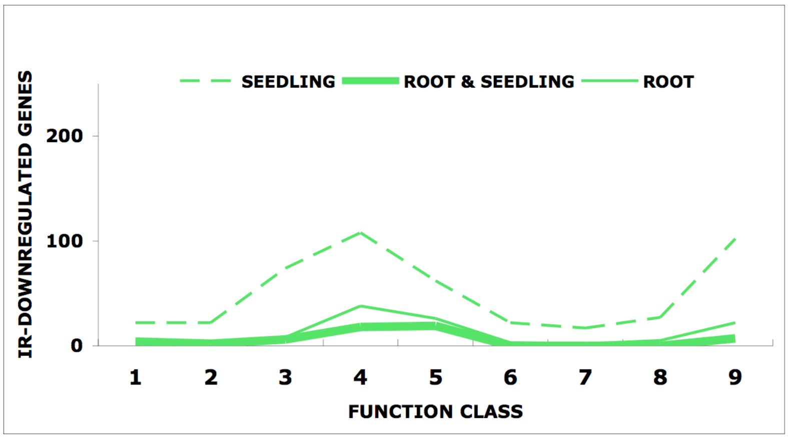
SEEDLING
ROOT & SEEDLING
ROOT
IR-DOWNREGULATED GENES
0
100
200
1 2 3 4 5 6 7 8 9
FUNCTION CLASS
